# High‐Throughput Discovery of a Rhombohedral Twelve‐Connected Zirconium‐Based Metal‐Organic Framework with Ordered Terephthalate and Fumarate Linkers

**DOI:** 10.1002/anie.202108150

**Published:** 2021-11-16

**Authors:** Adam M. Tollitt, Rebecca Vismara, Luke M. Daniels, Dmytro Antypov, Michael W. Gaultois, Alexandros P. Katsoulidis, Matthew J. Rosseinsky

**Affiliations:** ^1^ Department of Chemistry University of Liverpool Liverpool L69 7ZD UK

**Keywords:** chemical space, high-throughput synthesis, Metal-organic frameworks, ordered linkers, rhombohedral distortion

## Abstract

We report a metal‐organic framework where an ordered array of two linkers with differing length and geometry connect [Zr_6_(OH)_4_O_4_]^12+^ clusters into a twelve‐connected **fcu** net that is rhombohedrally distorted from cubic symmetry. The ordered binding of equal numbers of terephthalate and fumarate ditopic carboxylate linkers at the trigonal antiprismatic Zr_6_ core creates close‐packed layers of fumarate‐connected clusters that are connected along the single remaining threefold axis by terephthalates. This well‐defined linker arrangement retains the three‐dimensional porosity of the Zr cluster‐based UiO family while creating two distinct windows within the channels that define two distinct guest diffusion paths. The ordered material is accessed by a restricted combination of composition and process parameters that were identified by high‐throughput synthesis.

## Graphical abstract

The ordered arrangement of terephthalate and fumarate linkers over the **fcu** net produces a zirconium‐based MOF Zr_6_(BDC)_3_(Fum)_3_ with two distinct windows and multiple guest diffusion pathways. This phase is formed by single‐step synthesis in a narrow region of the available chemical space, and its identification required a large set of synthetic experiments that were facilitated by automated high‐throughput methods.

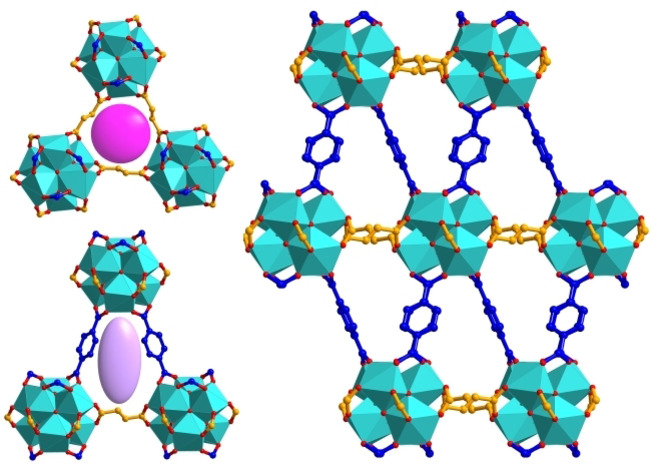

## Introduction

Metal‐organic frameworks, MOFs, are crystalline and porous materials composed of metal‐based nodes and organic linkers.^[1]^ They demonstrate extensive structural diversity and tunability due to the plethora of available choices for and arrangements of these building blocks.^[2]^ Many key MOF structural families^[3]^ arise from systematically expandable topologies that are defined by the chemistry and geometry of the interaction between a single node and a single linker. These topologies generate the structures for applications^[4]^ that depend on the pore geometries and dimensionalities, arising from the number, nature and connectivity of windows, channels and cages in the materials. The positionally ordered introduction of multiple linkers into the network topologies of these key families offers a distinct mechanism for precise control of the guest‐accessible space. The associated requirement for extra linker components can be expected to complicate full exploration of the resulting larger chemical space.

Automated high‐throughput (HT) methods for materials synthesis and analysis are powerful tools for the systematic exploration of multiparameter chemical systems.^[5]^ They enable the screening of larger numbers of reactions than purely manual methods, accelerating the detailed investigation of complex chemical spaces. The large numbers of variables associated with MOF synthesis and in particular with multiple linker MOFs renders HT synthesis an appropriate approach for the discovery of new phases.^[6]^

**Figure 1:**
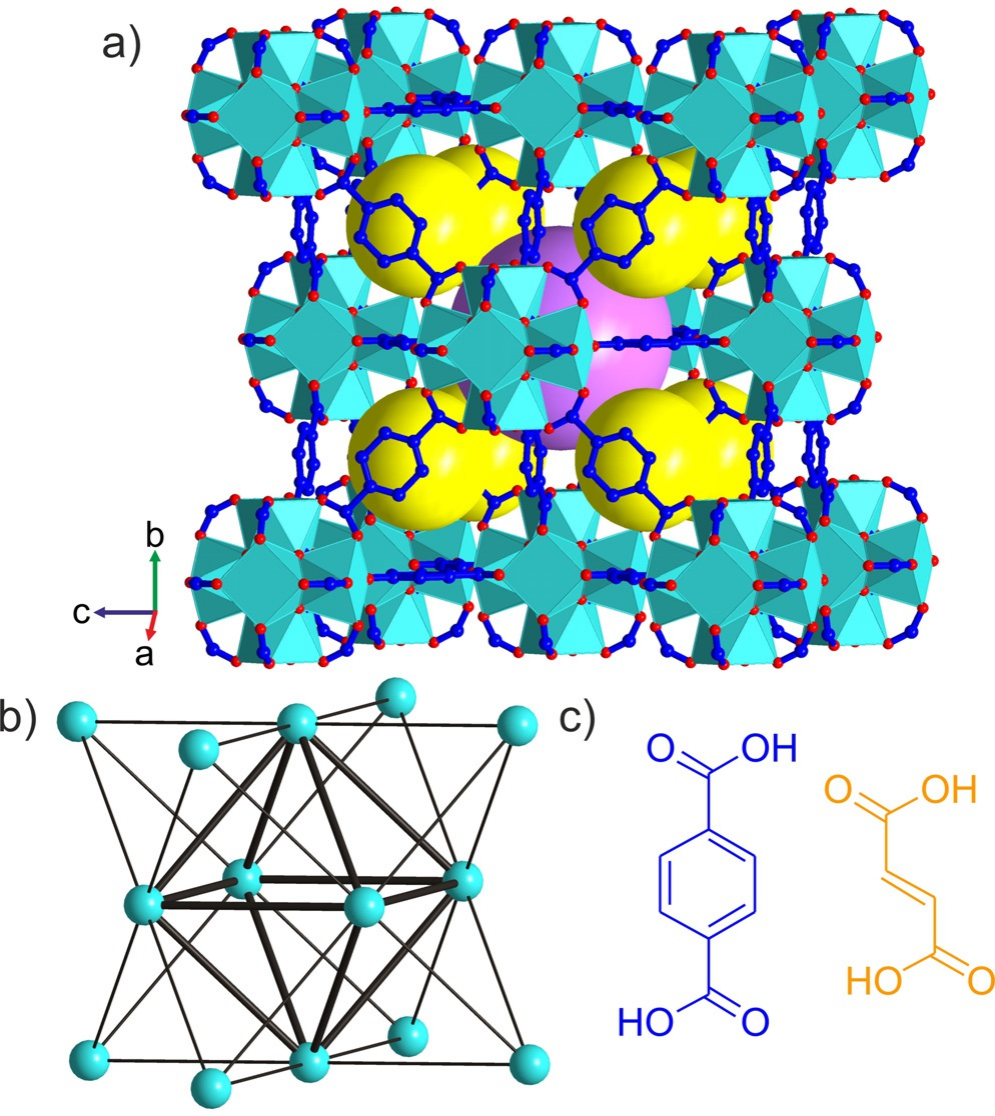
a) Crystal structure of UiO‐66 with [Zr_6_O_4_(OH)_4_]^12+^ clusters connected by terephthalate linkers. The yellow and purple spheres represent the guest‐accessible regular tetrahedral and octahedral cages, respectively. Zr cyan, O red, C blue; H (C−H, O−H) not shown. b) Simplified representation of UiO‐66 as a 12‐connected **fcu** net, where the cyan vertices correspond to the inorganic [Zr_6_O_4_(OH)_4_]^12+^ clusters and the edges to the organic linkers. A regular octahedron of individual clusters (thick edges) is surrounded by eight regular tetrahedra of clusters, and connected to them by sharing one type of equilateral triangular face. These octahedral and tetrahedral cages define the porosity of UiO‐66. c) Terephthalic (blue) and fumaric (orange) acid are ditopic linkers of different shape and length that afford the **fcu** frameworks UiO‐66 shown in (a) and MOF‐801 (Figure S1), respectively.

Zirconium carboxylate MOFs built from the [Zr_6_O_4_(OH)_4_]^12+^ cluster unit have received considerable attention not only for their high chemical stability but also for their three‐dimensional porosity, structural diversity^[7]^ and ability to incorporate many chemical functionalities.^[8]^ The 12‐connected, 12‐c, framework of UiO‐66^[9]^ (Figure 1 a) with face‐centered cubic (**fcu**) topology is based on an octahedral Zr_6_ core whose 12 edges are bridged by ditopic carboxylate linkers to form the extended structure: the four μ_3_‐O^2−^ and four μ_3_‐OH^−^ ligands are located alternately above each triangular face of the core. The positions of the twelve carboxylate linkers around the inorganic cluster define a cuboctahedron. UiO‐66 has been the prototype for the development of several mixed‐linker MOFs. Cubic structures of randomly distributed linkers with the same lengths are produced either by single‐step synthetic protocols^[10]^ or by postsynthetic linker exchange reactions.^[11]^ Also the use of linkers with different lengths in single‐step reactions affords disordered cubic structures.^[12]^ Alternatively, materials where linkers of different lengths are ordered to decorate the framework in a well‐defined manner have been synthesised by sequential installation of linkers.^[13]^ This relies on the initial preparation of an 8‐c framework with **bcu** topology where only eight of the twelve edges of the Zr_6_ octahedron are bridged by linkers. A subsequent synthetic step then introduces four linkers onto the remaining edges.^[14]^ This narrows the synthetic space to afford ordered mixed‐linker materials, but necessarily constrains the composition to a 2:1 ratio of the first to the second linker. It also restricts the accessible ordered two‐linker structures, because the stepwise assembly gives the parent structure decisive influence on the outcome. For example, the porosity is always characterised by one type of window between the tetrahedral and octahedral cages. Reflecting these limits, there has been no report of an **fcu** Zr MOF with ordered multiple linkers obtained from a single‐step synthesis, or of any such material with a linker ratio distinct from 2:1 or a structural arrangement of linkers different from 8 plus 4. The aim of this study is single‐step self‐assembly of an ordered array of two linkers that is not subject to these constraints.

We present a new 12‐c Zr MOF with equal content of ordered terephthalate and fumarate linkers. This material was prepared in a single‐step synthesis and discovered by high‐throughput exploration of the chemical space defined by the reagents ZrOCl_2_/terephthalic acid/fumaric acid/formic acid in DMF. The material is based on the underlying **fcu** topology where the [Zr_6_O_4_(OH)_4_]^12+^ clusters are connected by six terephthalate and six fumarate linkers. This ordered arrangement of the two distinct linkers produces trigonally distorted tetrahedral and octahedral cages which share two types of windows that are differentiated by the linkers that describe them. This is the first **fcu** Zr MOF with two windows and reflects the opportunity to control three‐dimensional MOF porosity precisely through multiple linker chemistry.

## Results and Discussion

The ditopic linkers terephthalate and fumarate were selected for the exploration of mixed‐linker Zr MOF synthesis as they both form 12‐c **fcu** topology MOFs, UiO‐66 and MOF‐801^[15]^ (Figure S1), respectively, and they differ in length (the distance between carboxylate carbons is 6.0 and 3.9 Å, respectively) and shape (linear versus zig‐zag, Figure 1 c). These distinct linker geometries can confer structural diversity on the products.

**Figure 2:**
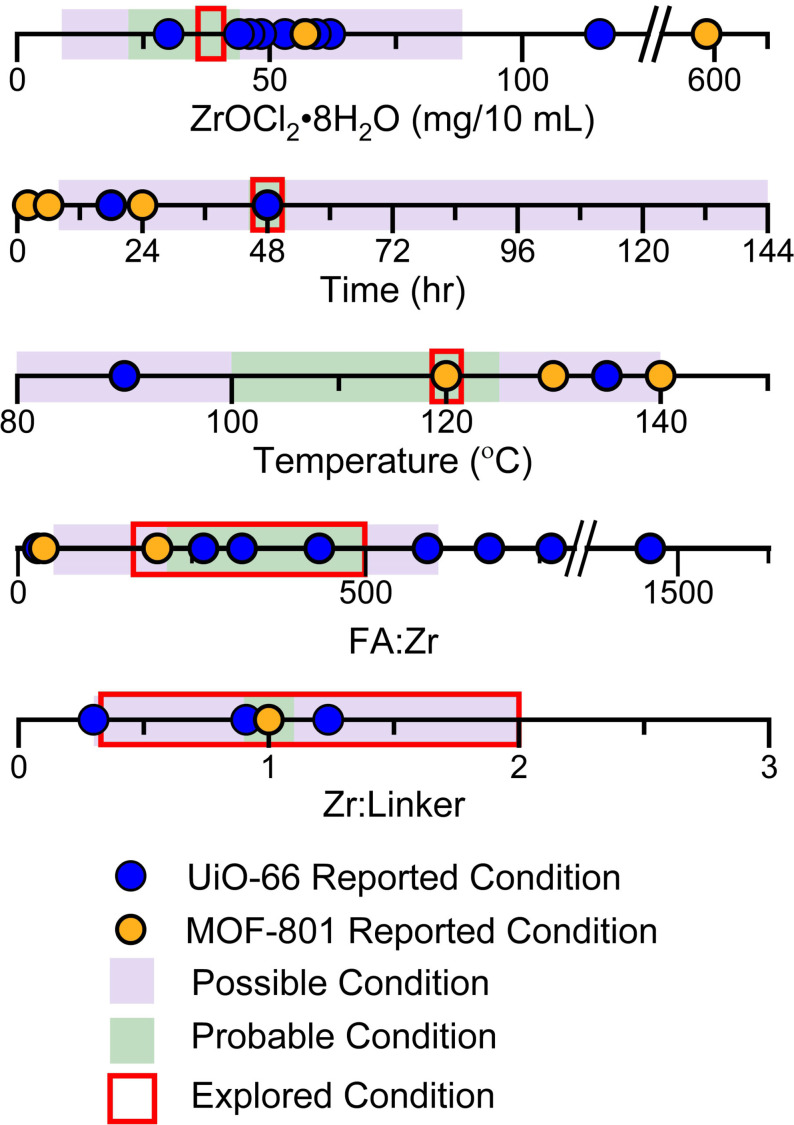
Summary of reported conditions from the literature for the synthesis of UiO‐66 (9 reported conditions (Table S5), identified by blue symbols), and MOF‐801 (5 reported conditions (Table S6), identified by yellow symbols) with ZrOCl_2_⋅8 H_2_O as the metal source and formic acid (FA) as the modulator. These conditions, and the requirements of the liquid‐handling robot for full solubility of reagents at room temperature, in contrast to literature studies that might use both solid and liquid reagents, were used to select the range of conditions explored for the synthesis of a Zr‐based MOF with mixed terephthalate and fumarate linkers. Conditions in which one would reasonably expect to obtain a crystalline product given the constraints of this specific chemistry on the robotic platform used are classed as “preferred” (indicated by the region highlighted in green), whilst those where the formation of a crystalline product within the accessible chemistry here is seen as less likely, are classed as “considered” (indicated by the regions highlighted in purple). The regions outlined in red indicate the range over which each of these variables was explored in this work. The time and temperature used were fixed at 48 hours and 120 °C respectively, as was the identity of the solvent, DMF, and modulator, formic acid. The amount of ZrOCl_2_⋅8 H_2_O used was limited by its solubility in DMF and fixed at 38 mg/10 mL.

The synthesis of UiO‐66^[16]^ and MOF‐801^[17]^ has been achieved over a broad range of overall concentrations and compositions of the starting materials in various solvent systems. We chose ZrOCl_2_⋅8 H_2_O as the metal source because it forms the stable solutions essential for automated dispensing. We then selected the reaction conditions to be explored for the robotic high‐throughput synthesis of Zr‐based MOF with mixed terephthalate (T) and fumarate (F) linkers by evaluating reported conditions from the literature where ZrOCl_2_⋅8 H_2_O was used (summarised in Figure 2 and Tables S5 and S6) in **fcu** MOF synthesis. The solvent, DMF, and modulator, formic acid (FA), were chosen to be the same for all reactions as they have been used extensively in synthesis of both UiO‐66 and MOF‐801. The time and temperature of reaction were fixed at 48 hours and 120 °C, respectively, to provide sufficient duration and high enough temperature to favour formation of a new phase without encountering problems due to DMF volatility. In order to exploit the liquid‐handling robot used to prepare reaction mixtures, we selected conditions that enabled the use of stock solutions of the reaction components. The implicit necessity for these solutions to be stable at room temperature throughout the duration of the automated sample preparation, which required up to 2 hours to complete, imposed a limit on the concentration of the ZrOCl_2_⋅8 H_2_O stock solution used of 0.0225 M, because of its solubility in DMF. The volume of this stock solution added to each reaction mixture was also fixed, giving each 10 mL‐scale reaction mixture the same ZrOCl_2_⋅8 H_2_O concentration of 38 mg/10 mL. The combinatorial parameters explored in the high‐throughput syntheses were thus limited to the ratio between the two linkers, (T:F), and their total amount relative to the quantity of Zr, Zr:(T+F), as well as the amount of formic acid (FA:Zr). These three parameters were expected to have the largest influence on the chemistry and potential formation of a new phase.^[18]^

**Figure 3:**
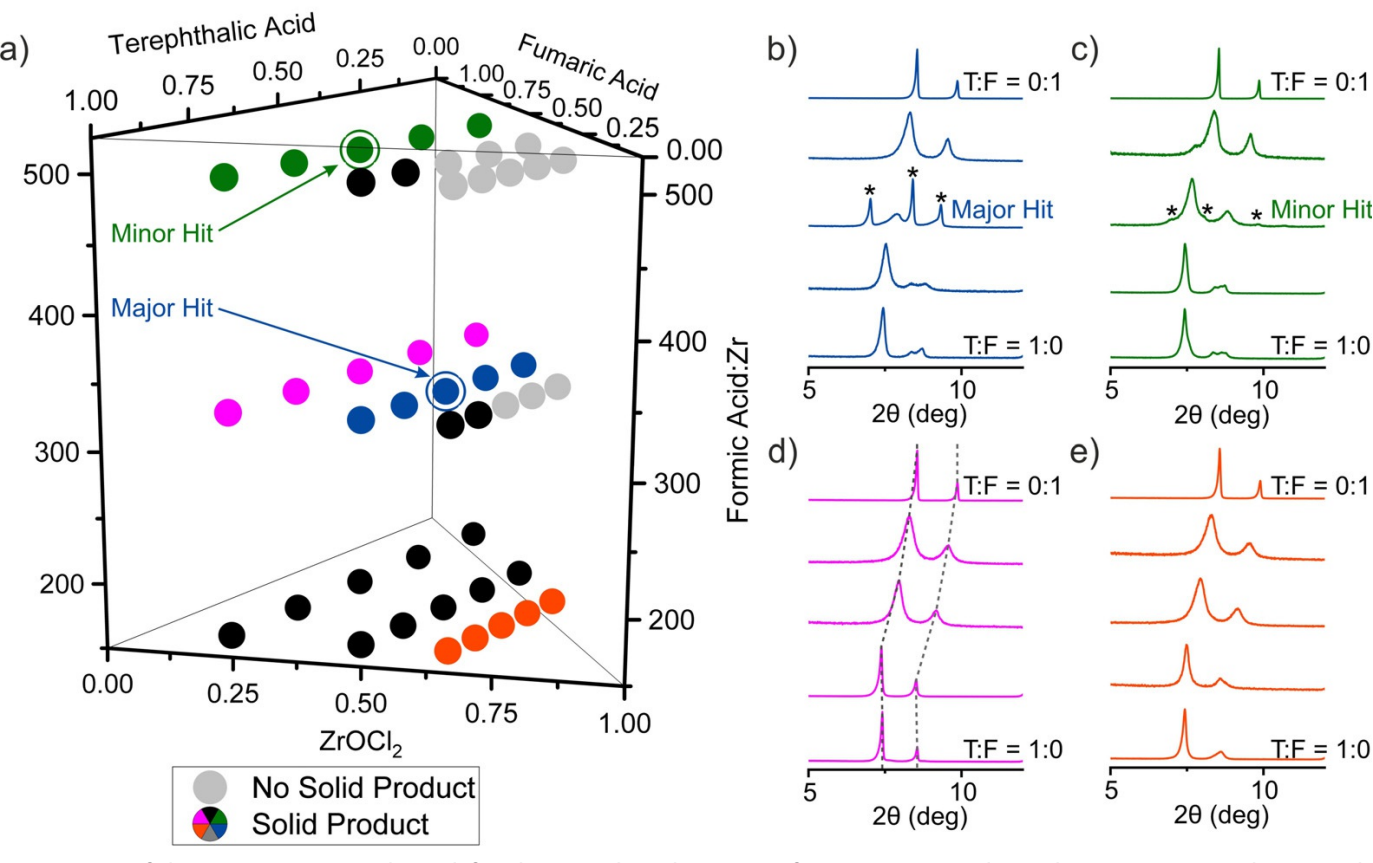
a) Compositions of the 45 reactions selected for the initial exploration of new compounds in the system ZrOCl_2_—terephthalic acid—fumaric acid—formic acid with DMF as solvent. 11 light grey symbols identify the reactions that lead to no solid product. The rest of the symbols are colour coded and correspond to the PXRD patterns in panels (b)–(e) and, for the 14 samples with black symbols, in Figure S2. b)–e) PXRD patterns of 20 representative samples arranged in sets of 5. Within each coloured set of samples the only variable is the T:F ratio, the bottom pattern corresponds to pure terephthalic acid as the linker and the top to pure fumaric acid. All patterns contain peaks that correspond to cubic phases with lattice parameter varying with T:F ratio (Figure S8). The dashed lines in (d) mark the shift of these peaks to higher 2*θ* angles as the T:F ratio decreases—this shift occurs in all the sets of patterns shown. The two circled points in (a) correspond to the compositions that provided the major (blue) and minor (green) hits in the search for a new phase. The extra diffraction features that indicate the presence of a new phase, corresponding to these hits, are indicated by asterisks.

We adopted a grid search and used automated dispensing of reaction mixtures by liquid‐handling robots to accelerate the exploration of the space. The chemical space was sub‐divided to span a broad range of compositions, covered by a set of 45 points (Table S2), in the first, broad iteration of the exploration of new phases. The two linkers were introduced in mixtures of five different compositions, T:F=1:0, 0.75:0.25, 0.5:0.5, 0.25:0.75 and 0:1, at each of three molar ratios between ZrOCl_2_ and total amount of linker Zr:(T+F)=0.25:0.75, 0.5:0.5, and 0.66:0.33 (Figure 3 a). Formic acid was employed as modulator in three different amounts FA:Zr=167, 334, 501. The components of each reaction mixture were transferred to a vial by automated dispensing of their solution in DMF except formic acid, which was dispensed neat. DMF was added to each of the reaction mixtures to give each vial the same fill factor, with a total volume of reaction of 10 mL. All of the reaction mixtures were prepared in parallel, with the reaction components added with the same order of addition (formic acid—ZrOCl_2_—T:F stock—DMF), and were run under the same condition (120 °C for 48 hours).

After the completion of the reactions, the samples were classified by visual inspection. Only 11 out of 45 (light grey in Figure 3 a) yielded no solid product. PXRD showed that each of the 34 solid products is crystalline. The patterns of the samples prepared with only one linker confirmed the presence of the known phases of UiO‐66 for terephthalate and MOF‐801 for fumarate (Figures S3 and S7) under the HT reaction conditions used in this study. The mixed‐linker samples of the reaction sets displayed in red and purple in Figure 3 a display diffraction peaks at low angle that correspond only to those of the cubic phases. Their diffraction patterns were fitted to cubic unit cells with lattice parameters between the cubic end‐members MOF‐801 and UiO‐66 (Figures S4–S6 and S8). These samples can be characterised as single phase solid solution terephthalate/fumarate Zr MOFs similar to those reported recently by Zhou.^[12]^ The presence of both linkers in these samples has been verified by ^1^H NMR analysis on the digested solids (Figures S9–S11), where the molar ratio T:F is higher than the nominal value, for example a solid synthesised with T:F=1:1 contains T:F=1:0.64 (Table S8).

The PXRD pattern from the sample with composition Zr:T:F=0.5:0.25:0.25 and FA:Zr=334, marked as the circled blue point in Figure 3 a, exhibits three sharp peaks that do not correspond to any of the known phases of the studied system and cannot be indexed with the cubic cell (Figure 3 b). The sample Zr:T:F=0.25:0.375:0.375 and FA:Zr=501, marked as the circled green point in Figure 3 a, also presents the same peaks with much lower intensity (Figure 3 c). These two results were identified as hits, major and minor, respectively, in the search for a new phase in the present system and guided the continued exploration in a second, focussed library that explored a smaller volume of the chemical space.

**Figure 4:**
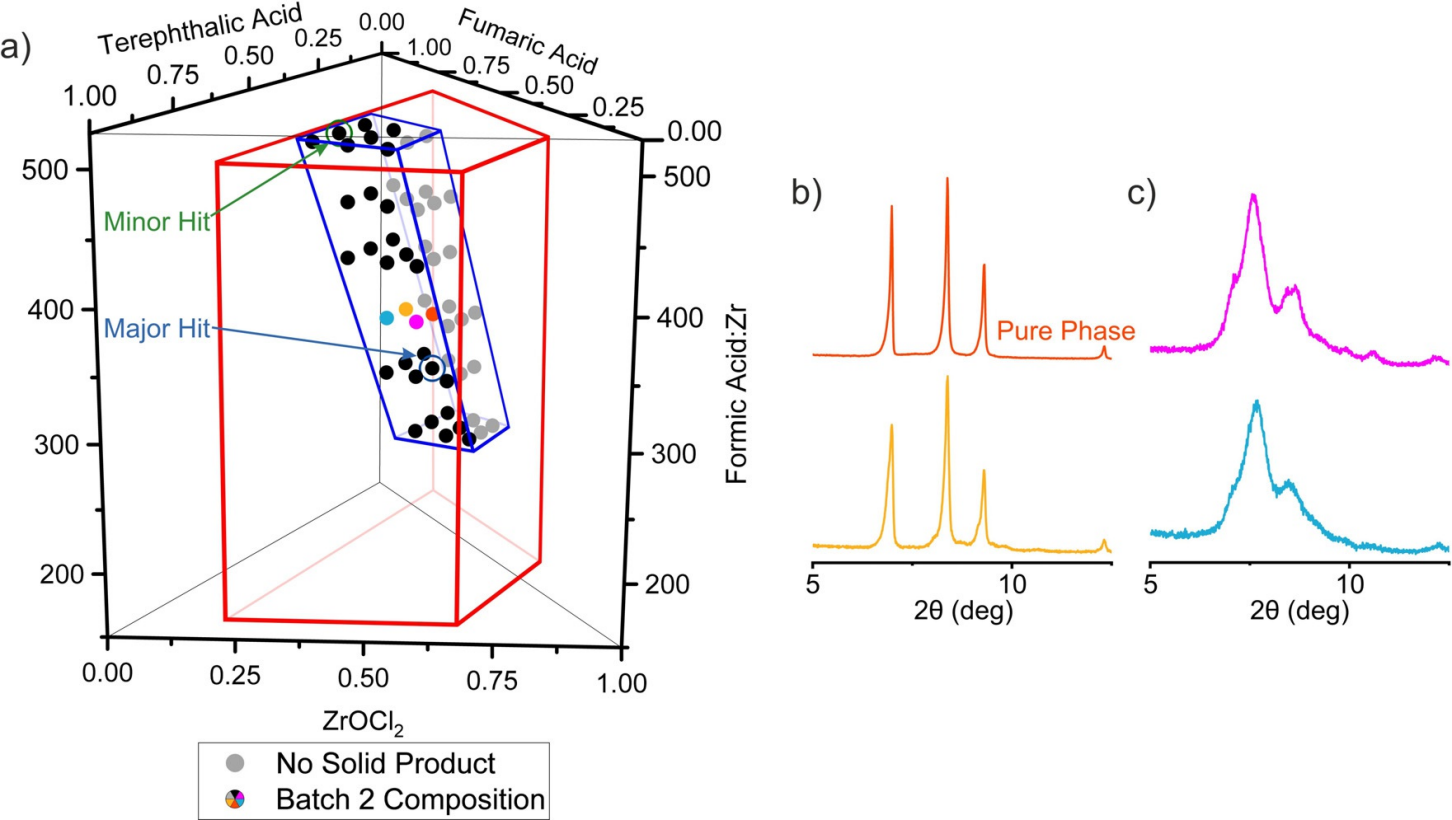
a) Compositions of the 54 reactions selected for the second iteration exploration of the system ZrOCl_2_—terephthalic acid—fumaric acid—formic acid with DMF as solvent. The major and minor hits from the first batch (Figure 3) guided the selection of the second batch. The area bound by the red trapezoid represents the chemical space explored in the first batch of reactions, whilst the area outlined in blue represents the space explored in the second batch. The grey points identify reactions that did not yield a solid product. b) PXRD patterns of four samples synthesised at FA:Zr=376 and different Zr:T:F ratios. The orange and yellow patterns corresponds to samples synthesized with T:F=0.5:0.5 and show the three main peaks of the new phase. Purple and blue patterns correspond to samples with T:F=0.625:0.375 ratio and show peaks of the solid solution phases adopting the cubic structures of the parents. The orange pattern corresponds to the pure form of the new phase.

The second iteration of reactions was focused in a region of the chemical space (Figure 4 a) that was selected to include the two hits from the first batch and to have its centre closer to the major than the minor hit. This volume was fifteen times smaller (Supplementary Note 2 and Figure S12 in the Supporting Information) than that described by the first set of samples, and was more densely covered with 54 reaction compositions. The linker molar ratio, T:F, ranges from 0.375:0.625 to 0.625:0.375 because both hits were obtained from reaction mixtures with equimolar amounts of linkers. The FA:Zr ratio ranged from 292 to 501, divided into six selected values. For each of these six FA:Zr ratios, the Zr:(T+F) molar ratio adopted three values (Table S4). The rest of the reaction conditions (use of DMF as solvent, temperature 120 °C for 48 hours, size of vials 20 mL and the execution protocol with the robot remained) exactly the same as in the first batch.

As observed in the first batch, in the second batch there were distinct regions where the reactions yielded no solid product, which were the mixtures with low linker and high modulator content, and regions where solid product was formed. PXRD patterns of the solid products (Figures 4 and S13) showed that the target new phase was observed only in the samples prepared with an equimolar ratio of the two linkers (Figure 4 b), while the rest of the samples displayed diffraction peaks that correspond to the solid solution phases (Figure 4 c). This noticeable difference between samples prepared with T:F=0.5:0.5 and T:F=0.625:0.375 demonstrates the requirement for large number of experiments, facilitated by high‐throughput methods, to isolate the new phase even within the narrowed chemical space of the second batch, and emphasises the contrast in precision of conditions required to form this phase with the broad region over which the linker‐disordered cubic phases form (Figure S14). The sample with composition Zr:T:F=0.5:0.25:0.25 and FA:Zr=376 exhibits the pure form of the new phase. Zr_6_(BDC)_3_(Fum)_3_ crystallizes in *R* $\bar{3}$ (space group no. 148), *a*=*b*=12.69646(7) Å, *c*=37.9733(4) Å, *V*=5301.20(8) Å^3^. The space group assignment was based on the observed systematic absences. Structure solution was carried out using synchrotron PXRD data collected at beamline I11 (Diamond Light Source, *λ*=0.826596(10) Å) through a combined Monte Carlo/Simulated Annealing approach with TOPAS‐Academic V5, followed by Rietveld refinement (see Characterisation Techniques and Figure S16). During refinement, the occupancies of the linkers were fixed at the values determined by ^1^H NMR analysis of the activated material (Figure S23), while the occupancies of MeOH molecules located in the pores were obtained from refinement.

**Figure 5:**
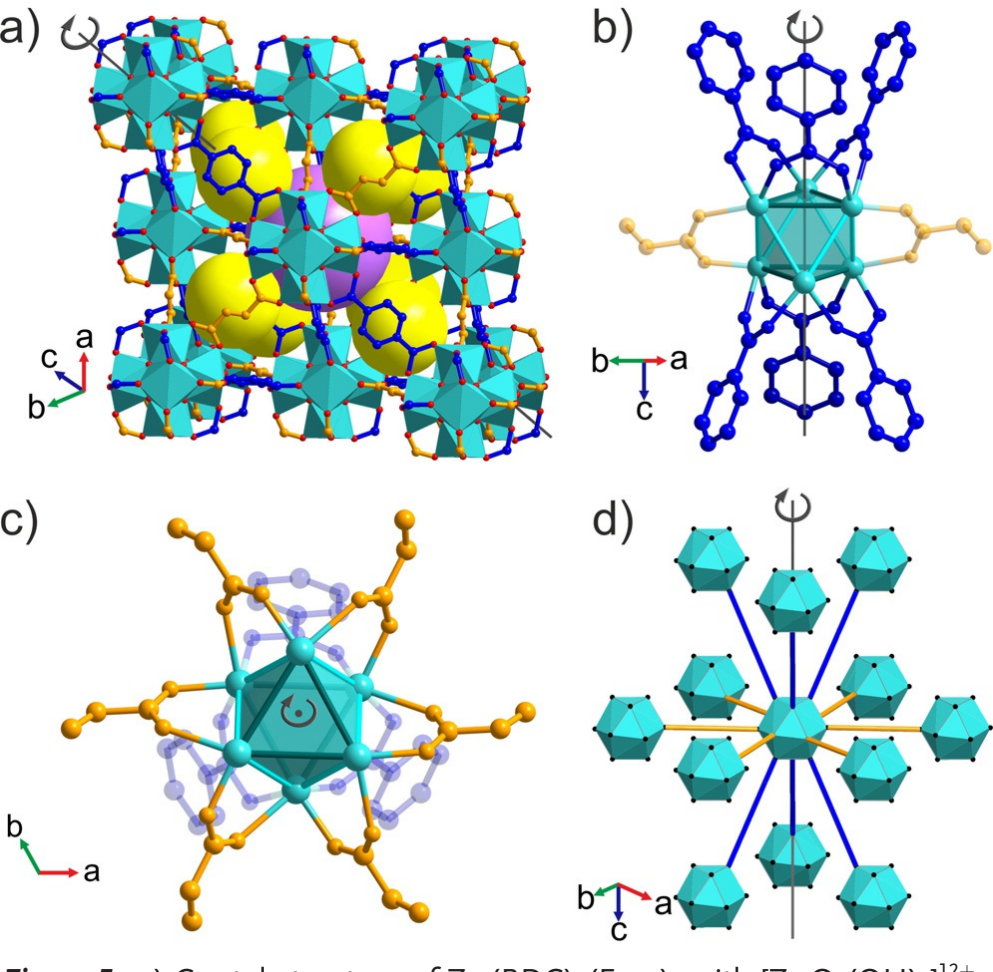
a) Crystal structure of Zr_6_(BDC)_3_(Fum)_3_ with [Zr_6_O_4_(OH)_4_]^12+^ clusters (cyan, O in red) connected by terephthalate (blue) and fumarate (orange) linkers. Yellow and purple spheres represent the centres of the distorted tetrahedral (trigonal pyramid) and octahedral (trigonal antiprism) cages, respectively. b) Terephthalate linkers (blue) occupy the edges of the two equilateral triangular faces of the Zr_6_ trigonal antiprisms that are aligned with the threefold axis of the rhombohedral structure. The Zr–Zr distance defining these edges is 3.523(6) Å (c) Fumarate linkers (orange) occupy the remaining six edges of the antiprism, with Zr–Zr distances of 3.460(3) Å, which connect the equilateral triangular faces (only two of the linkers are shown for clarity in (b)). The terephthalate‐bridged edges of the Zr_6_ trigonal antiprism are rendered in a darker colour in (b) and (c). d) The distorted Zr_6_O_4_(OH)_4_(COO)_12_ cuboctahedra defined by the ligand oxygen positions are arranged in fcc packing, where three close‐packed layers in the ABC sequence are shown, viewed perpendicular to the threefold axis. Each cuboctahedron is connected by the long blue edges (terephthalates) to six clusters in the layers above and below its layer, and by the short orange edges (fumarates) to six other clusters in the same layer. The close‐packed fumarate‐only layers that define the *ab* plane are stacked along the unique threefold axis of the rhombohedral cell.

Zr_6_(BDC)_3_(Fum)_3_ is a 12‐c framework of [Zr_6_O_4_(OH)_4_]^12+^ clusters connected by six terephthalates and six fumarates in the **fcu** topology (Figure 5 a). The Zr_6_ core of the cluster adopts trigonal antiprismatic geometry, where the two equilateral triangular faces align with the unique threefold axis of the rhombohedral structure. The edges of these equilateral triangular faces are occupied in an ordered manner only by the terephthalate linkers (Figures 5 b and S17a), which connect with six other clusters, three in the close‐packed layer above and three in the layer below (Figure 5 d). The remaining six edges of the cluster are occupied by fumarates (Figures 5 c and S17b) that connect to six other clusters in a hexagonal planar fashion—these are the neighbours within the close‐packed layer occupied by the cluster itself (Figures 5 d and S18). The six remaining faces of the trigonal antiprism Zr_6_ core are then isosceles triangles described by one terephthalate‐bridged and two fumarate‐bridged edges.

**Figure 6:**
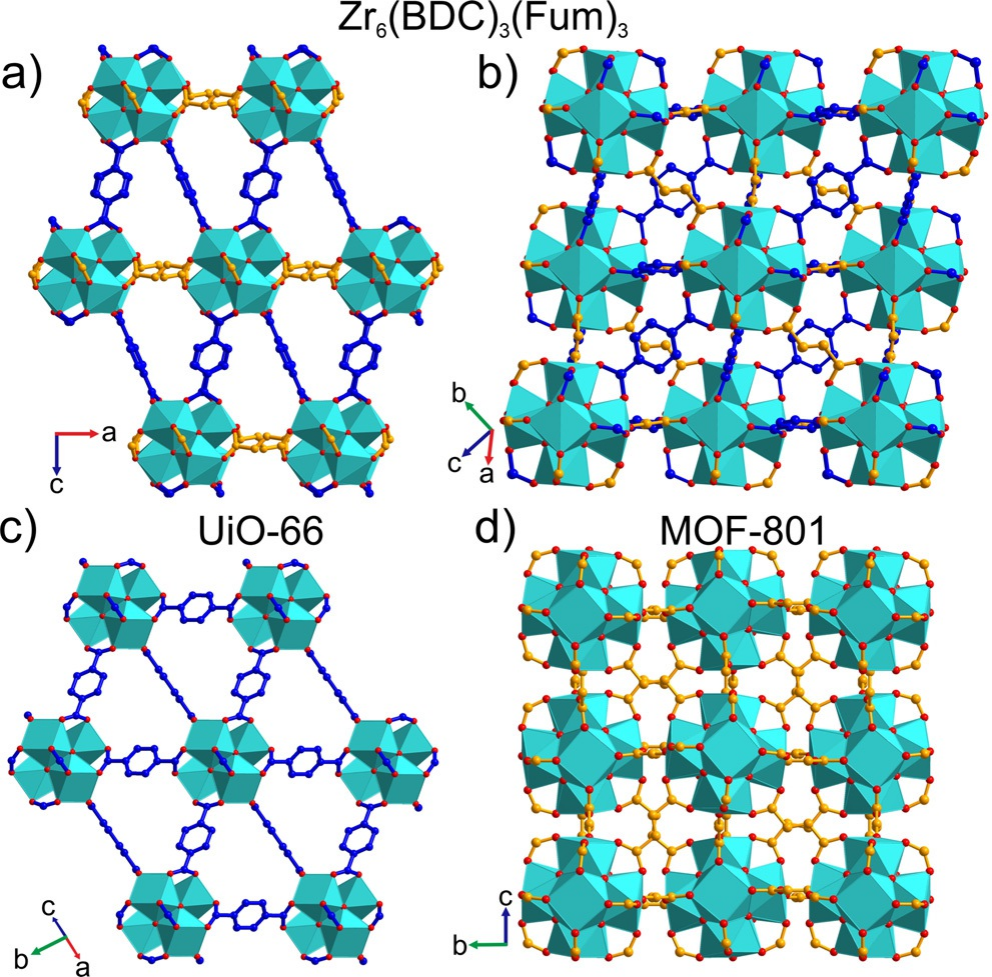
Comparison of (a), (b) the crystal structure of Zr_6_(BDC)_3_(Fum)_3_, viewing (a) the [010] and (b) [10‐13] planes, with (c) UiO‐66, viewing the [110] plane, and (d) MOF‐801, viewing the [100] plane. The *ab* plane of Zr_6_(BDC)_3_(Fum)_3_ (a) is contracted when compared with the equivalent plane in UiO‐66 (c), because it is defined by fumarate, rather than terephthalate, linkers. As in UiO‐66, the [Zr_6_O_4_(OH)_4_]^12+^ clusters of Zr_6_(BDC)_3_(Fum)_3_ all have the same orientation (b). This contrasts with their arrangement in MOF‐801 (d), where the zig‐zag shape of the fumarate linkers causes the clusters to tilt in alternating directions about the intercluster vectors that define the unit cell directions. Each Zr in Zr_6_(BDC)_3_(Fum)_3_ has a distorted square antiprismatic coordination environment, as the oxygens from fumarate and terephthalate adopt different bond lengths to the Zr centre (Figure S19). This is reflected in the distinction between the square faces defined by the ligand oxygens in (d), and the distortion of this square into two edge‐sharing triangles in (b).

The lower rhombohedral symmetry of Zr_6_(BDC)_3_(Fum)_3_ (Figure 6 a) compared to the cubic *Fm* $\bar{3}$ *m* structure of UiO‐66 (Figure 6 c) is associated with this ordered arrangement of the two linkers. The shorter length of fumarates compared to terephthalates shrink the intercluster distances in the *ab* plane in comparison with those that have a *c* axis component and induces the rhombohedral distortion (Figures 5 d, 6 c and S18). Thus, the unit cell volume per formula unit of Zr_6_(BDC)_3_(Fum)_3_, 1767 Å^3^, lies in between those of UiO‐66 (2231 Å^3^) and MOF‐801 (1418 Å^3^). In contrast to MOF‐801, where the zigzag shape of the fumarates causes the alternating tilting of the Zr clusters about the unit cell vectors (Figure 6 d), the clusters of Zr_6_(BDC)_3_(Fum)_3_ adopt the same orientations (Figure 6 b), resulting in asymmetric binding of the fumarates to the Zr centres (Figure S19), with Zr–O_Fumarate_ bond lengths of 1.964(13) and 2.363(4) Å. The terephthalates are essentially symmetric bridges, with Zr–O_Terephthalate_ bond lengths 2.148(2) and 2.079(4) Å.

**Figure 7:**
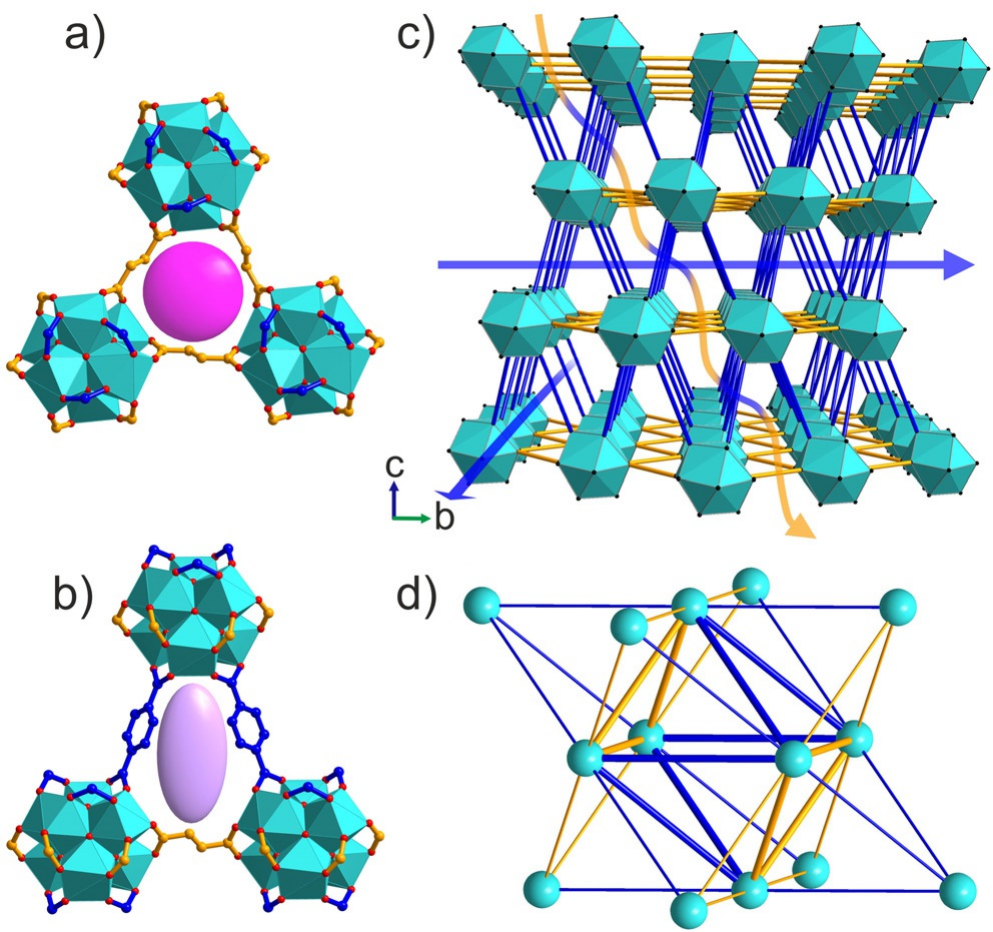
Zr_6_(BDC)_3_(Fum)_3_ has two types of triangular windows, one (a) is equilateral with all three sides composed of fumarates (3F) and the other (b) is isosceles composed of two terephthalates and one fumarate (2T1F). The pink sphere and purple ellipsoid highlight the difference in the shape of these two windows created by the ordered two‐linker arrangement. c) The crystal structure of Zr_6_(BDC)_3_(Fum)_3_ viewed perpendicular to the *c* axis, with Zr_6_O_4_(OH)_4_(COO)_12_ cuboctahedra in cyan and terephthalate and fumarate linkers represented by orange and blue lines. Diffusion paths parallel to the *ab* plane (demonstrated by the blue arrows) pass through only the 2T1F windows, whereas diffusion paths involving transport along the *c* axis (demonstrated by the blue‐and‐yellow arrow) have to pass through both 2T1F and 3F window types. d) Simplified representation of Zr_6_(BDC)_3_(Fum)_3_ as a distorted **fcu** net. Cyan vertices correspond to the inorganic [Zr_6_O_4_(OH)_4_]^12+^ clusters and the blue and yellow edges to terephthalate and fumarate linkers. The ratio of the edge lengths is blue:yellow=1.15. The tetrahedral and octahedral cages share two types of faces, an equilateral triangle (3F, three yellow edges) and an isosceles triangle (2T1F, two blue and one yellow edge).

Zr clusters and fumarates thus form close‐packed hexagonal layers, describing the *ab* plane of the rhombohedral structure, which are connected by the terephthalates (Figure 7 c) in the third dimension. This regular arrangement of two linkers with different lengths on the **fcu** net defines the unique shapes of the cages and windows, and controls the details of the three‐dimensional pore system. The tetrahedral cage adopts a trigonal pyramidal shape and the octahedral cage has a trigonal antiprismatic shape (Figure S20 a and b). These distorted tetrahedral and octahedral cages are connected by two types of triangular windows, one fully composed of fumarates, 3F, (Figure 7 a) and one composed of two terephthalates and one fumarate, 2T1F, (Figure 7 b). In contrast to the known Zr **fcu** MOF structures that all have one type of window, Zr_6_(BDC)_3_(Fum)_3_ has two distinct types of window between the cages (Figure 7 d). The degree of rhomdohedral distortion in Zr_6_(BDC)_3_(Fum)_3_ is expressed in the different distances between opposing windows of the same type in the octahedral cage, measured through the cage centre. This distance between 3F windows is 12.650(2) Å and between 2T1F windows is 10.555(3) Å (Figure S20 c and d).

The two windows in Zr_6_(BDC)_3_(Fum)_3_ define two types of possible diffusion pathways for guest molecules. In the path parallel to the Zr fumarate layers, the guest passes through the 2T1F window only (blue arrow in Figure 7 c), whereas in any path that involves motion out of this plane, the guest passes through both windows (the blue‐and‐yellow arrow in Figure 7 c). There is no diffusion pathway involving exclusively 3F windows. This can be easily demonstrated by the net representation (Figure 7 d), where any pathway entering a tetrahedral cavity through the 3F window (three yellow edges) will have to continue through one of the three 2T1F windows (two blue and one yellow edge).

**Figure 8:**
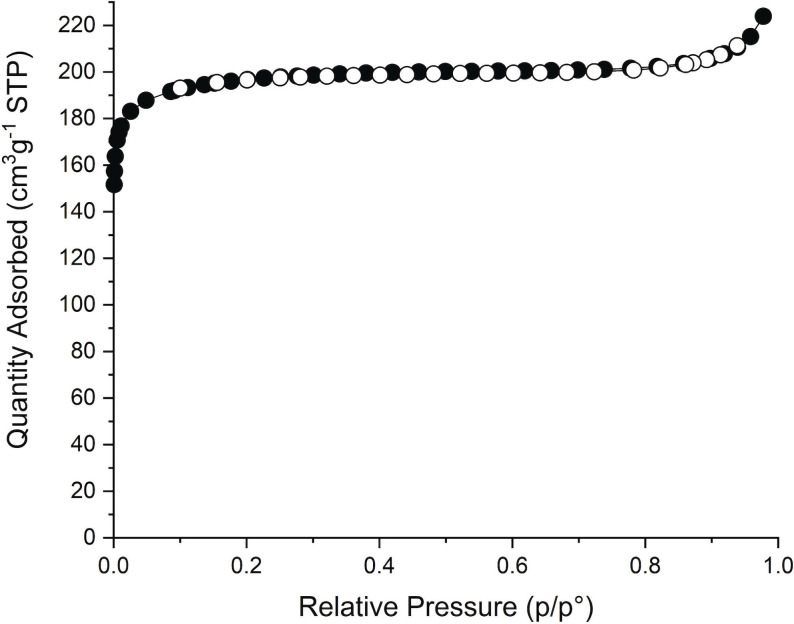
N_2_ adsorption desorption isotherm at 77 K of Zr_6_(BDC)_3_(Fum)_3_, which has a BET surface area of 783 m^2^ g^−1^ and pore volume of 0.32 cm^3^g^−1^. The closed symbols correspond to the adsorption branch and the open symbols to the desorption branch.

Compositional and porous characterisation of Zr_6_(BDC)_3_(Fum)_3_ was performed on a sample prepared from synthesis that involves ZrCl_4_ as starting material because this affords the product at higher yield, 83 %, than the ZrOCl_2_‐based synthesis, 15 %. (Experimental protocols Supporting information). The phase purity of this sample was confirmed with PXRD (Figure S24). The organic components of Zr_6_(BDC)_3_(Fum)_3_ were analysed by ^1^H NMR after digestion of the sample in NaOD/D_2_O. The MeOH‐exchanged sample displays molar ratios T:F:MeOH:FA=1:0.92:3.4:0.2 (Figure S22). The deviation from the equimolar composition between the two linkers and the presence of formates in this sample are indicative of missing linker defects in the Zr_6_(BDC)_3_(Fum)_3_ structure. It is well known that modulated synthesis of Zr MOFs promotes the formation of defects,^[19]^ which are predominantly missing linkers and occasionally missing clusters.^[20]^ It has been demonstrated that single crystals of UiO‐66 contain 10 % of missing linkers,[^16a^, ^20a^] whereas powders of UiO‐66 prepared with trifluoroacetate modulator contain 33 % of missing linkers.^[21]^ Solvent exchange with MeOH produces pairs of MeO^−^/MeOH as terminal ligands on the defect sites of UiO‐66.^[22]^ To provide a more accurate framework composition of Zr_6_(BDC)_3_(Fum)_3_, the MeOH exchanged sample was activated under high vacuum at 60 °C to remove non‐coordinating MeOH from the pores before digestion and ^1^H NMR analysis (Figure S23), while the structure of the material is preserved (Figure S24). The molar ratio T:F:MeOH:FA=1:0.92:0.6:0.2 is used to derive the formula of the activated material as Zr_6_O_4_(OH)_4_(BDC)_2.77_(Fum)_2.55_(MeO)_0.83_(MeOH)_0.83_‐ (Formate)_0.55_, in which 0.68 linkers, or 11.3 % of the 6 linkers in the idealised composition, are missing, with their sites in each cluster occupied by formate and pairs of MeO^−^/MeOH ligands. These missing linkers have an effect on the porous properties of the material, as they generate extra accessible space and decrease the density of the framework. Zr_6_(BDC)_3_(Fum)_3_ exhibits a type I N_2_ adsorption desorption isotherm (Figure 8) with a BET surface area of 783 m^2^ g^−1^ and a pore volume of 0.32 cm^3^g^−1^. Both experimental values are larger than the theoretical values, 714 m^2^ g^−1^ and 0.26 cm^3^g^−1^ respectively calculated using Zeo++ (see Supplementary Note 4 in the Supporting Information).^[23]^

The experimental BET surface area and pore volume of Zr_6_(BDC)_3_(Fum)_3_ lie between the respective values of MOF‐801 (690 m^2^ g^−1^/ 0.27 cm^3^ g^−1^) and UiO‐66 (1290 m^2^ g^−1^/0.49 cm^3^ g^−1^).^[17a]^ The ordered arrangement of two ditopic linkers in the **fcu** net controls the global porous properties, surface area and pore volume, as well as the local ones, the size and the shape of the individual cages and windows. This approach offers additional tuning capabilities for the porous properties of a high symmetry net through the precise locally‐ and long‐range ordered definition of intermediate surface areas created from distinct pore shapes. The well‐established isoreticular expansion or contraction of a single linker MOF offer large changes in surface area and pore size that preserve the shape and number of cages and windows,^[24]^ while solid solution mixed‐linker MOFs generate intermediate surface areas in locally heterogeneous pore systems arising from long‐range multiple linker disorder.^[12]^

## Conclusion

The chemical space defined by ZrOCl_2_, terephthalic acid, fumaric acid and formic acid was explored to identify new MOF by high‐throughput experimental screening. The two‐linker ordered MOF, Zr_6_(BDC)_3_(Fum)_3_, was the only new phase discovered and it is formed in a narrow region of this space, in contrast to the linker‐disordered cubic material formed by the same two linkers. The identification of the linker‐ordered system by single‐step self‐assembly then required the screening at fine compositional resolution that is enabled by the high‐throughput approach. Although each linker individually affords an important, well‐studied member of the key **fcu** net Zr‐based MOF family, both with one pore window and a single guest diffusion path, Zr_6_(BDC)_3_(Fum)_3_ is not a simple intermediate between these structures. Rather, its structure is generated by ordered linker decoration of the **fcu** net that breaks the symmetry to introduce anisotropy into the three‐dimensional porosity, which is now characterised by two distinct diffusion paths. The ordering of terephthalate and fumarate binding to the [Zr_6_O_4_(OH)_4_]^12+^ cluster creates two windows of different shape that describe the distorted octahedral and tetrahedral cages defining these paths. This ordering precisely defines the porosity locally to each cage and is distinct from the locally heterogeneous tuning offered by disordered multiple linker MOF average structures. The resulting simultaneous tuning of pore size and shape, which affords interval pore volume between the parents, differs from isoreticular expansion in that it tunes within a defined range of extra‐framework space. Multiple linker ordered decoration of canonical single linker MOF topologies can harness the resulting combinatorial and chemical diversity of linker sets to generate new porous materials families where the size and shape of the internal space can be precisely modified for optimal guest interaction.

### Associated Content

The Supporting information file contains detailed experimental procedures and results from PXRD, ^1^H NMR and TGA measurements. It also contains additional figures, tables and notes. Deposition Numbers 2089846 contain the supplementary crystallographic data for this paper. These data are provided free of charge by the joint Cambridge Crystallographic Data Centre and Fachinformationszentrum Karlsruhe Access Structures service www.ccdc.cam.ac.uk/structures. The datasets supporting the findings of this study are available from the University of Liverpool and can be found at https://datacat.liverpool.ac.uk/id/eprint/1460.

## Conflict of interest

The authors declare no conflict of interest.

## Supporting information

As a service to our authors and readers, this journal provides supporting information supplied by the authors. Such materials are peer reviewed and may be re‐organized for online delivery, but are not copy‐edited or typeset. Technical support issues arising from supporting information (other than missing files) should be addressed to the authors.

Supporting Information
