# Supplementary material for: High‐Throughput Discovery of a Rhombohedral Twelve‐Connected Zirconium‐Based Metal‐Organic Framework with Ordered Terephthalate and Fumarate Linkers
Source: Angew Chem Int Ed Engl. 2021 Nov 16;60(52):26939–46. doi: 10.1002/anie.202108150 (PMC9299659; doi:10.1002/anie.202108150)
Supplement: Supplementary file 1 — Supporting Information [file ANIE-60-26939-s001.pdf]

Angewandte Chemie
Eine Zeitschrift der Gesellschaft Deutscher Chemiker

Supporting Information

High-Throughput Discovery of a Rhombohedral Twelve-Connected Zirconium-Based Metal-Organic Framework with Ordered Terephthalate and Fumarate Linkers

*Adam M. Tollitt, Rebecca Vismara, Luke M. Daniels, Dmytro Antypov, Michael W. Gaultois, Alexandros P. Katsoulidis, and Matthew J. Rosseinsky**

anie_202108150_sm_miscellaneous_information.pdf

Supplementary Information:

Table of Contents

Experimental Protocols (Tables S1 – S4) S3

Characterisation Techniques S9

Crystal Structure of MOF-801 (Figure S1) S11

Summary of Literature Reported Conditions for the Synthesis of UiO-66 and MOF-801 (Tables S5 – S6) S12

Batch 1 PXRD Data (Figures S2 – S8 and Table S7) S13

Supplementary Note 1 .... S20
Batch 1 ^{1}H NMR Data (Figures S9 – S11 and Table S8) S21
Supplementary Note 2 (Figure S12) S24

Batch 2 PXRD Data (Figure S13) S26
Summary of All Reaction Compositions (Figure S14) S27
$Zr_6(BDC)_3(Fum)_3$ PXRD Indexing and Rietveld Refinement (Figures S15 – S16) S29
Crystallographic Data Table (Table S9) S31
Crystal Structure of $Zr_6(BDC)_3(Fum)_3$ (Figures S17 – S20) S32
Supplementary Note 3 .... S36
Calculation of Experimental Composition of $Zr_6(BDC)_3(Fum)_3$ by ^{1}H NMR (Figures S21 – S23) S37
PXRD of MeOH Exchanged and Activated $Zr_6(BDC)_3(Fum)_3$ (Figure S24) S40
Thermogravimetric Analysis (TGA) of Activated $Zr_6(BDC)_3(Fum)_3$ (Figure S25) S41
Elemental Analysis (Table S10) S42
Supplementary Note 4 .... S43
Porosity Measurement Data (Table S11) S43
PXRD $Zr_6(BDC)_3(Fum)_3$ in Water (Figure S26) S44
References S45

Experimental Protocols:

General Experimental Details:

All reagents were purchased from commercial suppliers and used without modification. Zirconyl chloride octahydrate ($ZrOCl_2$•$8H_2O$, purity 98%), zirconium chloride ($ZrCl_4$, anhydrous, purity 99.99%), fumaric acid (purity ≥99%), terephthalic acid (purity ≥98%), sodium hydroxide-d (40 wt. % in D_2O) and deuterium oxide (99.9 atom % D) were obtained from Sigma-Aldrich Co. N,N-Dimethylformamide (DMF) and methanol were obtained from Fisher Scientific. Formic acid (purity 97%) was obtained from Alfa Aesar. Automated liquid dispensation was performed with an Eppendorf epMotion 5075t liquid-handling platform. 20 mL headspace screw neck glass vials with metal screw caps were purchased from VWR International.

MOF synthesis with liquid-handling robots:

Mixed-linker Zr-based MOFs with terephthalate and fumarate linkers, were prepared in batches and the components of each reaction mixture were transferred to the reaction vessel, a 20 mL headspace screw neck glass vial, by automated dispensation of their solution in DMF, with the exception of formic acid, which was dispensed neat.

In a typical batch synthesis, $ZrOCl_2$•$8H_2O$ was dissolved in DMF to prepare a stock solution of concentration 0.0225 M. The concentration of this solution was fixed, and chosen to be as high as possible, whilst remaining below the solubility limit of $ZrOCl_2$•$8H_2O$ in DMF at room temperature to ensure that the solution remained as a stable homogeneous mixture for the entire duration of the automated sample preparation, which, for the second batch of syntheses in which 54 samples were prepared, required 2 hours to complete. The volume of 0.0225 M $ZrOCl_2$•$8H_2O$ stock solution dispensed into each reaction mixture, and therefore the quantity of $ZrOCl_2$•$8H_2O$ used in each 10 mL scale reaction was also fixed at 38 mg, 0.12 mmol. The linker stock solutions were prepared by dissolving terephthalic acid and fumaric acid in DMF at a total linker, (T+F), concentration of 0.15 M, with different T:F molar ratios (Table S1 and Table S3).

The different reaction mixtures were prepared using the Eppendorf epMotion 5075t liquid handling platform, with dispensation directly into the reaction vessel. In each batch of syntheses, the individual reaction mixtures were prepared in parallel and the components were added to the mixture in the same order (formic acid – $ZrOCl_2$•$8H_2O$ stock – T:F linker stock – DMF). DMF was dispensed into each vial to ensure every reaction had the same fill factor, with a total reaction solution volume of 10 mL. The vials were then sealed with metal screw caps, before being heated at 120 °C for 48 hours. Powder products were collected by centrifugation and washed with DMF and methanol.

In the specific examples below, batch 1 refers to samples from the first iteration and batch 2 to samples in the second iteration.

Batch 1 Experimental:
A stock solution of the metal source was prepared by dissolving $ZrOCl_2 \cdot 8H_2O$ (5.63 mmol, 1.81 g) in DMF (250 mL). Linker stock solutions with five different T:F molar ratios, T:F = 1:0, 0.75:0.25, 0.5:0.5, 0.25:0.75 and 0:1, were prepared at a total linker (T+F) concentration of 0.15 M in DMF (25 mL). The quantities of terephthalic acid and fumaric acid used in each solution are summarised in Table S1.

Table S1 Compositions, T:F, of the five different linker stock solutions used in the first batch of syntheses, and the quantities of terephthalic acid and fumaric acid used to prepare 0.15 M solutions of each in 25 mL of DMF.

**T:F**	**Terephthalic Acid**		**Fumaric Acid**	
**Molar Ratio**	**mmol**	**g**	**mmol**	**g**
1:0	3.75	0.62	0	0
0.75:0.25	2.81	0.47	0.94	0.11
0.5:0.5	1.88	0.31	1.88	0.22
0.25:0.75	0.94	0.16	2.81	0.33
0:1	0	0	3.75	0.44

Each of the 45 reaction mixtures in the first batch of syntheses (Table S2) were prepared in parallel, with automated dispensation of neat formic acid, followed by the $ZrOCl_2 \cdot 8H_2O$ stock solution and the T:F linker stock solution, with the corresponding T:F molar ratio for each composition, directly into the reaction vessel (20 mL headspace screw neck glass vial) in the quantities specified in Table S2. DMF was then dispensed into each vial to make the total volume of each reaction mixture 10 mL, thus giving each reaction vessel the same fill factor. The order in which the reaction components were added, formic acid – $ZrOCl_2 \cdot 8H_2O$ stock – T:F linker stock – DMF, was the same for each of the 45 individual reaction mixtures in the batch, with the process of automated dispensation lasting 1.5 hours. The vials were sealed with metal screw caps before being heated at 120 °C for 48 hours. Powder products were collected by centrifugation and washed twice with DMF (5 mL) followed by methanol (5 mL).

Table S2 Compositions of the 45 reaction mixtures selected for the first batch synthesis exploring the system $ZrOCl_2$ – terephthalic acid – fumaric acid – formic acid with DMF as the solvent. The outcome of each reaction is given with each composition classified by whether a solid product was obtained and, for those which yielded a solid product, the phase observed by PXRD (C – cubic; R – rhombohedral; M – multiple phases, cubic and rhombohedral). Compositions of the major and minor hits, highlighted in Figure 3, are highlighted in blue and green respectively.

Zr:T:F	FA:Zr	$ZrOCl_2 \cdot 8H_2O$ (mmol)	T (mmol)	F (mmol)	T:F (molar)	$ZrOCl_2 \cdot 8H_2O$ Stock (mL)	T:F Stock (mL)	FA (mL)	DMF (mL)	Solid? (Y/N)	Phase
**0.25:0:0.75**	167	0.12	0.00	0.36	0:1	5.29	2.40	0.75	1.56	Y	C
**0.25:0.19:0.56**	167	0.12	0.09	0.27	0.25:0.75	5.29	2.40	0.75	1.56	Y	C
**0.25:0.375:0.375**	167	0.12	0.18	0.18	0.5:0.5	5.29	2.40	0.75	1.56	Y	C
**0.25:0.56:0.19**	167	0.12	0.27	0.09	0.75:0.25	5.29	2.40	0.75	1.56	Y	C
**0.25:0.75:0**	167	0.12	0.36	0.00	1:0	5.29	2.40	0.75	1.56	Y	C
**0.5:0:0.5**	167	0.12	0.00	0.12	0:1	5.29	0.79	0.75	3.17	Y	C
**0.5:0.125:0.375**	167	0.12	0.03	0.09	0.25:0.75	5.29	0.79	0.75	3.17	Y	C
**0.5:0.25:0.25**	167	0.12	0.06	0.06	0.5:0.5	5.29	0.79	0.75	3.17	Y	C
**0.5:0.375:0.125**	167	0.12	0.09	0.03	0.75:0.25	5.29	0.79	0.75	3.17	Y	C
**0.5:0.5:0**	167	0.12	0.12	0.00	1:0	5.29	0.79	0.75	3.17	Y	C
**0.667:0:0.333**	167	0.12	0.00	0.06	0:1	5.29	0.40	0.75	3.57	Y	C
**0.667:0.083:025**	167	0.12	0.01	0.04	0.25:0.75	5.29	0.40	0.75	3.57	Y	C
**0.667:0.1665:0.1665**	167	0.12	0.03	0.03	0.5:0.5	5.29	0.40	0.75	3.57	Y	C
**0.667:0.25:0.083**	167	0.12	0.04	0.01	0.75:0.25	5.29	0.40	0.75	3.57	Y	C
**0.667:0.333:0**	167	0.12	0.06	0.00	1:0	5.29	0.40	0.75	3.57	Y	C
**0.25:0:0.75**	334	0.12	0.00	0.36	0:1	5.29	2.40	1.50	0.81	Y	C
**0.25:0.19:0.56**	334	0.12	0.09	0.27	0.25:0.75	5.29	2.40	1.50	0.81	Y	C
**0.25:0.375:0.375**	334	0.12	0.18	0.18	0.5:0.5	5.29	2.40	1.50	0.81	Y	C
**0.25:0.56:0.19**	334	0.12	0.27	0.09	0.75:0.25	5.29	2.40	1.50	0.81	Y	C
**0.25:0.75:0**	334	0.12	0.36	0.00	1:0	5.29	2.40	1.50	0.81	Y	C
**0.5:0:0.5**	334	0.12	0.00	0.12	0:1	5.29	0.79	1.50	2.42	Y	C
**0.5:0.125:0.375**	334	0.12	0.03	0.09	0.25:0.75	5.29	0.79	1.50	2.42	Y	C
**0.5:0.25:0.25**	**334**	**0.12**	**0.06**	**0.06**	**0.5:0.5**	**5.29**	**0.79**	**1.50**	**2.42**	**Y**	**R**
**0.5:0.375:0.125**	334	0.12	0.09	0.03	0.75:0.25	5.29	0.79	1.50	2.42	Y	C
**0.5:0.5:0**	334	0.12	0.12	0.00	1:0	5.29	0.79	1.50	2.42	Y	C
**0.667:0:0.333**	334	0.12	0.00	0.06	0:1	5.29	0.40	1.50	2.82	Y	C
**0.667:0.083:025**	334	0.12	0.01	0.04	0.25:0.75	5.29	0.40	1.50	2.82	Y	C
**0.667:0.1665:0.1665**	334	0.12	0.03	0.03	0.5:0.5	5.29	0.40	1.50	2.82	N	–
**0.667:0.25:0.083**	334	0.12	0.04	0.01	0.75:0.25	5.29	0.40	1.50	2.82	N	–
**0.667:0.333:0**	334	0.12	0.06	0.00	1:0	5.29	0.40	1.50	2.82	N	–
**0.25:0:0.75**	501	0.12	0.00	0.36	0:1	5.29	2.40	2.25	0.06	Y	C
**0.25:0.19:0.56**	501	0.12	0.09	0.27	0.25:0.75	5.29	2.40	2.25	0.06	Y	C
**0.25:0.375:0.375**	**501**	**0.12**	**0.18**	**0.18**	**0.5:0.5**	**5.29**	**2.40**	**2.25**	**0.06**	**Y**	**M**
**0.25:0.56:0.19**	501	0.12	0.27	0.09	0.75:0.25	5.29	2.40	2.25	0.06	Y	C
**0.25:0.75:0**	501	0.12	0.36	0.00	1:0	5.29	2.40	2.25	0.06	Y	C
**0.5:0:0.5**	501	0.12	0.00	0.12	0:1	5.29	0.79	2.25	1.67	N	–
**0.5:0.125:0.375**	501	0.12	0.03	0.09	0.25:0.75	5.29	0.79	2.25	1.67	N	–
**0.5:0.25:0.25**	501	0.12	0.06	0.06	0.5:0.5	5.29	0.79	2.25	1.67	N	–
**0.5:0.375:0.125**	501	0.12	0.09	0.03	0.75:0.25	5.29	0.79	2.25	1.67	Y	C
**0.5:0.5:0**	501	0.12	0.12	0.00	1:0	5.29	0.79	2.25	1.67	Y	C
**0.667:0:0.333**	501	0.12	0.00	0.06	0:1	5.29	0.40	2.25	2.07	N	–
**0.667:0.083:025**	501	0.12	0.01	0.04	0.25:0.75	5.29	0.40	2.25	2.07	N	–
**0.667:0.1665:0.1665**	501	0.12	0.03	0.03	0.5:0.5	5.29	0.40	2.25	2.07	N	–
**0.667:0.25:0.083**	501	0.12	0.04	0.01	0.75:0.25	5.29	0.40	2.25	2.07	N	–
**0.667:0.333:0**	501	0.12	0.06	0.00	1:0	5.29	0.40	2.25	2.07	N	–

Batch 2 Experimental:
A stock solution of the metal source was prepared by dissolving $ZrOCl_2•8H_2O$ (6.75 mmol, 2.18 g) in DMF (300 mL). Linker stock solutions with three different T:F molar ratios, T:F = 0.375:0.625. 0.5:0.5 and 0.625:0.375, were prepared at a total linker (T+F) concentration of 0.15 M in DMF (250 mL). The quantities of terephthalic acid and fumaric acid used in each solution are summarised in Table S3.

Table S3 Compositions, T:F, of stock solutions used in the second batch synthesis and the quantities of terephthalic acid and fumaric acid used to prepare 0.15 M solutions of each in 250 mL of DMF.

T:F	Terephthalic Acid		Fumaric Acid	
Molar Ratio	mmol	g	mmol	g
0.625:0.375	23.44	3.89	14.06	1.63
0.5:0.5	18.75	3.12	18.75	2.18
0.375:0.625	14.06	2.34	23.44	2.72

Each of the 54 individual reaction mixtures in the second batch of syntheses (Table S4) were prepared in parallel with automated dispensation neat formic acid, followed by the $ZrOCl_2•8H_2O$ stock solution and the T:F linker stock solution, with the corresponding T:F molar ratio for each composition, directly into the reaction vessel (20 mL headspace screw neck glass vial) in the quantities specified in Table S4. DMF was then dispensed into each vial to make the total volume of each reaction mixture 10 mL, thus giving each reaction vessel the same fill factor. The order in which the reaction components were added, formic acid – $ZrOCl_2•8H_2O$ stock – T:F linker stock – DMF, was the same for each of the 45 individual reaction mixtures in the batch, with the process of automated dispensation lasting 2 hours. The vials were sealed with metal screw caps before being heated at 120 °C for 48 hours. Powder products were collected by centrifugation and washed twice with DMF (5 mL) followed by methanol (5 mL). The sample which exhibited the $Zr(BDC)_3(Fum)_3$ phase purely was obtained with a yield of 15%.

Table S4 Compositions of the 54 reaction mixtures selected for the second iteration of the batch synthesis exploring the system $ZrOCl_2$ – terephthalic acid – fumaric acid – formic acid with DMF as the solvent. The outcome of each reaction is given with each composition classified by whether a solid product was obtained and, for those which yielded a solid product, the phase observed by PXRD (C – cubic; R – rhombohedral; M – multiple phases, cubic and rhombohedral; U – unclassifiable). Compositions of the four points, including the point which gave a pure phase (red), highlighted in Figure 4 are highlighted in the corresponding colour.

Zr:T:F	FA:Zr	$ZrOCl_2•8H_2O$ (mmol)	T (mmol)	F (mmol)	T:F (molar)	$ZrOCl_2•8H_2O$ Stock (mL)	T:F Stock (mL)	FA (mL)	DMF (mL)	Solid? (Y/N)	Phase
**0.5:0.19:0.31**	292	0.12	0.04	0.07	0.375:0.625	5.29	0.79	1.31	2.61	Y	C
**0.5:0.25:0.25**	292	0.12	0.06	0.06	0.5:0.5	5.29	0.79	1.31	2.61	Y	R
**0.5:0.31:0.19**	292	0.12	0.07	0.04	0.625:0.375	5.29	0.79	1.31	2.61	Y	C
**0.574:0.160:0.266**	292	0.12	0.03	0.06	0.375:0.625	5.29	0.59	1.31	2.81	N	–
**0.574:0.213:0.213**	292	0.12	0.04	0.04	0.5:0.5	5.29	0.59	1.31	2.81	Y	M
**0.574:0.266:0.160**	292	0.12	0.06	0.03	0.625:0.375	5.29	0.59	1.31	2.81	Y	U
**0.63:0.139:0.231**	292	0.12	0.03	0.04	0.375:0.625	5.29	0.47	1.31	2.93	N	–
**0.63:0.185:0.185**	292	0.12	0.03	0.03	0.5:0.5	5.29	0.47	1.31	2.93	N	–
**0.63:0.231:0.139**	292	0.12	0.04	0.03	0.625:0.375	5.29	0.47	1.31	2.93	Y	U
**0.4286:0.2143:0.3571**	334	0.12	0.06	0.1	0.375:0.625	5.29	1.06	1.5	2.16	Y	C
**0.4286:0.2857:0.2857**	334	0.12	0.08	0.08	0.5:0.5	5.29	1.06	1.5	2.16	Y	R
**0.4286:0.3571:0.2143**	334	0.12	0.1	0.06	0.625:0.375	5.29	1.06	1.5	2.16	Y	U
**0.5:0.1875:0.3125**	334	0.12	0.04	0.07	0.375:0.625	5.29	0.79	1.5	2.42	N	–
**0.5:0.25:0.25**	334	0.12	0.06	0.06	0.5:0.5	5.29	0.79	1.5	2.42	Y	R
**0.5:0.3125:0.1875**	334	0.12	0.07	0.04	0.625:0.375	5.29	0.79	1.5	2.42	Y	U
**0.574:0.160:0.266**	334	0.12	0.03	0.06	0.375:0.625	5.29	0.59	1.5	2.63	N	–
**0.574:0.213:0.213**	334	0.12	0.04	0.04	0.5:0.5	5.29	0.59	1.5	2.63	N	–
**0.574:0.266:0.160**	334	0.12	0.06	0.03	0.625:0.375	5.29	0.59	1.5	2.63	Y	C
**0.4286:0.2143:0.3571**	376	0.12	0.06	0.1	0.375:0.625	5.29	1.06	1.69	1.97	N	–
**0.4286:0.2857:0.2857**	**376**	**0.12**	**0.08**	**0.08**	**0.5:0.5**	**5.29**	**1.06**	**1.69**	**1.97**	**Y**	**R**
**0.4286:0.3571:0.2143**	**376**	**0.12**	**0.1**	**0.06**	**0.625:0.375**	**5.29**	**1.06**	**1.69**	**1.97**	**Y**	**C**
**0.5:0.19:0.31**	376	0.12	0.04	0.07	0.375:0.625	5.29	0.79	1.69	2.23	N	–
**0.5:0.25:0.25**	**376**	**0.12**	**0.06**	**0.06**	**0.5:0.5**	**5.29**	**0.79**	**1.69**	**2.23**	**Y**	**R**
**0.5:0.31:0.19**	**376**	**0.12**	**0.07**	**0.04**	**0.625:0.375**	**5.29**	**0.79**	**1.69**	**2.23**	**Y**	**C**
**0.574:0.160:0.266**	376	0.12	0.03	0.06	0.375:0.625	5.29	0.59	1.69	2.44	N	–
**0.574:0.213:0.213**	376	0.12	0.04	0.04	0.5:0.5	5.29	0.59	1.69	2.44	N	–
**0.574:0.266:0.160**	376	0.12	0.06	0.03	0.625:0.375	5.29	0.59	1.69	2.44	N	–
**0.333:0.250:0.417**	418	0.12	0.09	0.15	0.375:0.625	5.29	1.59	1.88	1.25	Y	C
**0.333:0.335:0.335**	418	0.12	0.12	0.12	0.5:0.5	5.29	1.59	1.88	1.25	Y	R
**0.333:0.417:0.250**	418	0.12	0.15	0.09	0.625:0.375	5.29	1.59	1.88	1.25	Y	C
**0.4286:0.2143:0.3571**	418	0.12	0.06	0.1	0.375:0.625	5.29	1.06	1.88	1.78	N	–
**0.4286:0.2857:0.2857**	418	0.12	0.08	0.08	0.5:0.5	5.29	1.06	1.88	1.78	Y	M
**0.4286:0.3571:0.2143**	418	0.12	0.1	0.06	0.625:0.375	5.29	1.06	1.88	1.78	Y	U
**0.5:0.1875:0.3125**	418	0.12	0.04	0.07	0.375:0.625	5.29	0.79	1.88	2.05	N	–
**0.5:0.25:0.25**	418	0.12	0.06	0.06	0.5:0.5	5.29	0.79	1.88	2.05	N	–
**0.5:0.3125:0.1875**	418	0.12	0.07	0.04	0.625:0.375	5.29	0.79	1.88	2.05	Y	U
**0.333:0.250:0.417**	460	0.12	0.09	0.15	0.375:0.625	5.29	1.59	2.06	1.06	N	–
**0.333:0.335:0.335**	460	0.12	0.12	0.12	0.5:0.5	5.29	1.59	2.06	1.06	Y	R
**0.333:0.417:0.250**	460	0.12	0.15	0.09	0.625:0.375	5.29	1.59	2.06	1.06	Y	C
**0.4286:0.2143:0.3571**	460	0.12	0.06	0.1	0.375:0.625	5.29	1.06	2.06	1.59	N	–
**0.4286:0.2857:0.2857**	460	0.12	0.08	0.08	0.5:0.5	5.29	1.06	2.06	1.59	N	–
**0.4286:0.3571:0.2143**	460	0.12	0.1	0.06	0.625:0.375	5.29	1.06	2.06	1.59	Y	C
**0.5:0.1875:0.3125**	460	0.12	0.04	0.07	0.375:0.625	5.29	0.79	2.06	1.86	N	–
**0.5:0.25:0.25**	460	0.12	0.06	0.06	0.5:0.5	5.29	0.79	2.06	1.86	N	–
**0.5:0.3125:0.1875**	460	0.12	0.07	0.04	0.625:0.375	5.29	0.79	2.06	1.86	N	–
**0.248:0.282:0.470**	501	0.12	0.14	0.23	0.375:0.625	5.29	2.4	2.25	0.06	Y	C
**0.248:0.376:0376**	501	0.12	0.18	0.18	0.5:0.5	5.29	2.4	2.25	0.06	Y	M
**0.248:0.470:0.282**	501	0.12	0.23	0.14	0.625:0.375	5.29	2.4	2.25	0.06	Y	U
**0.333:0.250:0.417**	501	0.12	0.09	0.15	0.375:0.625	5.29	1.59	2.25	0.88	Y	U
**0.333:0.335:0.335**	501	0.12	0.12	0.12	0.5:0.5	5.29	1.59	2.25	0.88	Y	U
**0.333:0.417:0.250**	501	0.12	0.15	0.09	0.625:0.375	5.29	1.59	2.25	0.88	Y	U
**0.4286:0.2143:0.3571**	501	0.12	0.06	0.1	0.375:0.625	5.29	1.06	2.25	1.41	N	–
**0.4286:0.2857:0.2857**	501	0.12	0.08	0.08	0.5:0.5	5.29	1.06	2.25	1.41	N	–
**0.4286:0.3571:0.2143**	501	0.12	0.1	0.06	0.625:0.375	5.29	1.06	2.25	1.41	Y	U

Synthesis of $Zr_6(BDC)_3(Fum)_3$ with $ZrCl_4$:
In order to produce larger amounts of sample required for the detailed characterisation of $Zr_6(BDC)_3(Fum)_3$ following structure solution, a point in the second batch of syntheses, with composition Zr:T:F = 0.4286:0.2857:0.2857 and FA:Zr = 334:1 was identified which yielded the $Zr_6(BDC)_3(Fum)_3$ phase with high purity and with good yield of 83%. A set of 12 identical reactions mixtures with this composition were prepared using $ZrCl_4$ as the metal source, in order to ensure the $Zr_6(BDC)_3(Fum)_3$ phase maximise the phase purity of the sample for detailed characterisation.
$ZrCl_4$ (0.12 mmol, 0.0277 g) was weighed into each vial before DMF (7.5 mL) and formic acid (1.5 mL) was added. The vials were sealed with metal screw caps and the reaction mixtures sonicated at 50 °C for 45 minutes. During this time, a linker stock solution, with the T:F molar ratio T:F = 0.5:0.5, was prepared at a total linker (T+F) concentration of 0.16 M by dissolving terephthalic acid (2 mmol, 0.3294 g) and fumaric acid (2 mmol, 0.2301 g) in DMF (25 mL). After sonication, 1 mL of the T:F linker stock solution was added to each of the reaction mixtures, and the vials resealed with metal screw caps, before being heated at 120 °C for 48 hours. The material was collected by centrifugation, with each reaction yielding 25 mg (83%). After the completion of the reaction, the purity of the solid product formed was determined by PXRD of the powder in the supernatant (Figure S24).
Solvent Exchange and Activation:
Reaction mixtures from the scale-out syntheses described above with $ZrCl_4$, which yielded the $Zr_6(BDC)_3(Fum)_3$ phase with high purity were combined and the material collected by centrifugation. The supernatant was decanted, replaced with DMF (25 mL) and the mixture shaken for 1 hour. This process of washing with DMF was repeated three times. The DMF was then decanted after centrifugation, and the material stirred in methanol (20 mL) for a total of 9 days, during which time the methanol was replaced 4 times. Each time the methanol was replaced, the material was collected by centrifugation, solvent decanted and the material allowed to dry under atmospheric conditions to the point that it can be handled as a powder. At this point of each exchange, a ^{1}H NMR was obtained after digestion of a small amount of the material in NaOD (60 μL) and D_2O (640 μL).
The methanol exchanged material, which exhibited no phase change by PXRD after the solvent exchange, giving the $Zr_6(BDC)_3(Fum)_3$ purely, and which did not show evidence for the presence of DMF decomposition products by NMR, was activated under high-vacuum (10^{-5} bar) at 60 °C for 24 hours to remove all guest species. The activated material was transferred to the sample tube for porosity measurement under an Ar atmosphere.

Characterisation Techniques:

Nuclear Magnetic Resonance (NMR) spectroscopy. All ^{1}H NMR spectra were recorded in solution using a Bruker ADVANCE-400 MHz NMR spectrometer. All spectra were recorded in a solvent mixture of D_2O (640 μL) and NaOD (60 μL), with the residual solvent peak of D_2O (4.79 ppm for ^{1}H).

Thermal analysis. Thermal Gravimetric Analysis (TGA) was performed under air atmosphere on a TA Instruments Q600 between 25 and 1000 °C, with a scan rate of 10 °C min^{-1} and gas flow of 100 mL min^{-1}.

CHN Microanalysis. Carbon, Hydrogen and Nitrogen content was determined by microanalytical procedures using an Elementar MicroCube CHN Analyser.

Gas sorption analysis. Nitrogen adsorption-desorption isotherm was collected at 77 K using a Micrometrics Tristar II PLUS Surface Area and Porosity Analyzer. Guest species were removed from the pores of the material under dynamic vacuum as detailed in the experimental section, before being degassed on the analysis port for 2 hours at 77 K prior to measurement. BET area was calculated using the pressure range $0.008 < P/P0 < 0.05$, which was selected using the consistency criteria.

Laboratory PXRD. Laboratory based powder X-ray data were collected at room temperature on Bruker D8 Advance diffractometers with a monochromated Cu radiation source ($K\alpha_1$, λ = 1.5406 Å).

Synchrotron PXRD. Synchrotron powder X-ray diffraction data were collected at the I11 beam line at Diamond Light Source with λ = 0.826596(10) Å. Data were collected at room temperature using the Position Sensitive Detector (PSD, Mythen-2), with the samples sealed in borosilicate capillaries.

X-ray Powder Diffraction.

Comparison of the PXRD pattern of $Zr_6(BDC)_3(Fum)_3$ with those of UiO-66 and MOF-801 suggests that the new material adopts a different symmetry. As a consequence, an independent indexing procedure was performed. A standard peak search followed by profile fitting was used to estimate the low-to-medium-angle peak maximum positions, providing approximate unit cell parameters through the Singular Value Decomposition algorithm[1] as implemented in TOPAS-Academic V5.[2] The space group was assigned based on the observed systematic absences which were consistent with $R\bar{3}$. The structure solution was performed by a combined Monte Carlo/Simulated Annealing approach within TOPAS-Academic V5.

The crystallographically independent portion of the Zr_6O_8 cluster and of the terephthalate and fumarate linkers were modelled by rigid bodies through the z-matrix formalism. Then, a combined Monte Carlo/Simulated Annealing approach was also used to locate the solvent in the preliminary framework. In order to simulate the solvent, three methanol molecules were described as rigid bodies and their centre of mass positions and orientations were freely refined. The structure refinement was then carried out with the Rietveld method,[3] as implemented in TOPAS-Academic V5. Average values were initially assigned to bond distances and angles [Bond distances for the rigid body describing the linkers: C−C 1.36 Å; exocyclic C−C 1.45 Å; C−H 1.05 Å; C−O 1.40 Å] for both the linker and solvent. During the final Rietveld refinement stages, bond distances (except for the C-H distances) were allowed to refine within constraints [Bond distances for the rigid body describing the linkers: C−C 1.34-1.43 Å; exocyclic C−C min 1.40-1.60 Å; C−O 1.39-1.56 Å]. A ten-term Chebyshev polynomial function was used to model the background with three fixed broad peaks based on a Pseudo-Voigt function to model contributions to the scattering from structural disorder (*e.g.*, disordered solvent) and the borosilicate capillary used to contain the sample. These peaks were introduced first in a Pawley fit at angles of 3.99°, 12.35° and 23.12° and fixed throughout the Rietveld refinement. The Fundamental Parameters Approach[4] was used to model the peak profile. The occupancies of the terephthalate and fumarate rigid bodies were fixed throughout the refinement to be

consistent with the composition measured through ^{1}H NMR on the activated material, which showed that 11.3% of the linkers were missing, corresponding to an occupancy of 0.923 for terephthalate and 0.85 for fumarate. Such defects resulting from missing linkers in UiO-66 MOF structures are well-documented.[5] A refined, isotropic displacement parameter was assigned to the Zr atoms; the lighter O atoms coordinated to Zr were assigned isotropic displacement parameters that were 1.0 Å^2 larger. Isotropic displacement parameters of the atoms within the terephthalate and fumarate rigid bodies were fixed to 2.0 Å^2 to minimise the number of refined parameters. This model gave a reasonable fit to the data with R_{wp} = 3.356%. Close inspection of the observed Fourier map showed a slightly anisotropic distribution of electron density localised around the carboxylate oxygens on both the terephthalate and fumarate linkers (positions O41 and O42 for terephthalate and positions O61 and O62 for fumarate). Additional oxygen atoms were added to the model to fit this density and with occupancies inverse to those of the carboxylate oxygen in both the terephthalate and fumarate rigid bodies (positions O41b and O42b set to 0.077, and O61b and O62b set to 0.15). The positions of these additional oxygens refined stably to fit this electron density, yielding an improved fit to the data with R_{wp} = 3.194%. These additional oxygen sites, which have been observed previously in UiO-66 MOFs,[5c, 6] are associated with coordinating formate species, the presence of which is confirmed by ^{1}H NMR, and as such the occupancies of the carboxylate carbons in the terephthalate and fumarate rigid bodies (positions C4 and C6, respectively) are fixed at full occupancy. This yields a refined composition of $Zr_6O_4(OH)_4(BDC)_{2.77}(Fum)_{2.55}(Formate)_{1.362}$ with $(MeOH)_{0.709(3)}$ located in the pores which is comparable to the measured composition of $Zr_6O_4(OH)_4(BDC)_{2.77}(Fum)_{2.55}(MeO)_{0.83}(MeOH)_{0.83}(Formate)_{0.55}$. The final Rietveld refinement is shown in Figure S16, and refinement parameters are listed in Table S9. The pertinent CIF file is supplied as Electronic Supplementary Material and is deposited under CCDC 2089846.

Crystal Structure of MOF-801:

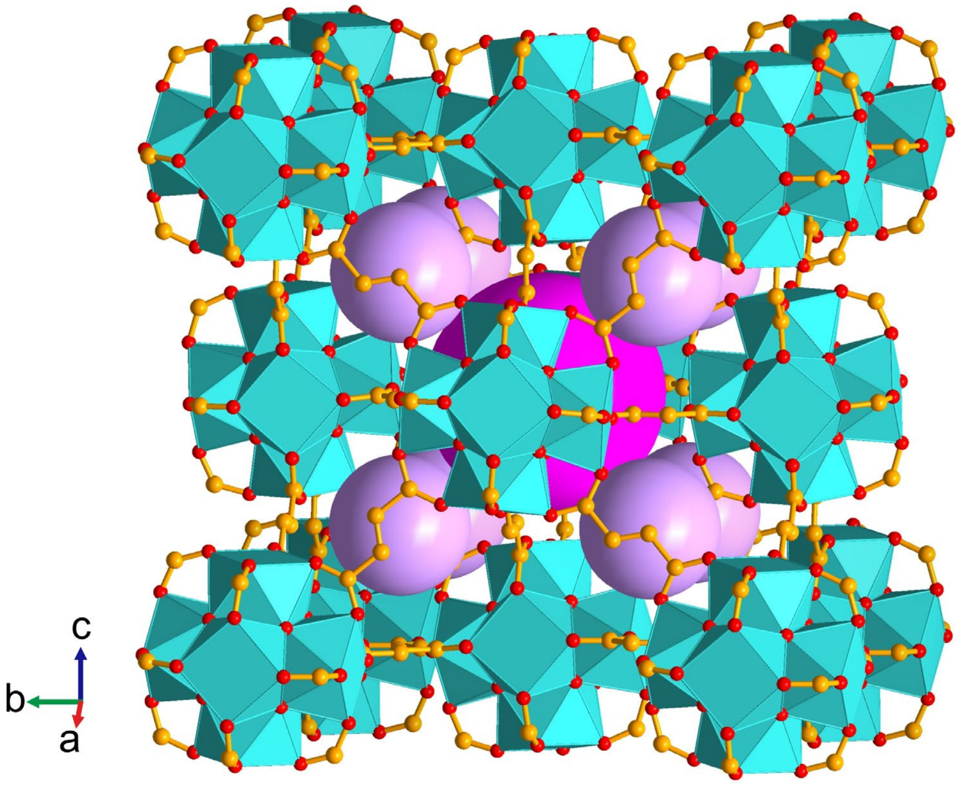

Figure S1 Crystal structure of MOF-801, where the $[Zr_6O_4(OH)_4]^{12+}$ clusters (cyan, O red) are connected by fumarate linkers (orange). The purple and pink spheres represent the space accessible in the tetrahedral and octahedral cages respectively.

Summary of Literature Reported Conditions for the Synthesis of UiO-66 and MOF-801:

Table S5 Summary of the nine different literature reported conditions for which the successful synthesis of UiO-66 has been achieved in DMF using $ZrOCl_2 \cdot 8H_2O$ as the metal source and formic acid (FA) as the modulator.[5c, 7] The quantity of $ZrOCl_2 \cdot 8H_2O$ presented in the table have been scaled to a total volume of reaction (DMF + FA) equal to 10 mL.

$ZrOCl_2 \cdot H_2O$ (mg/10 mL)	Time (hr)	Temperature (°C)	FA:Zr	Zr:Linker
**30.0**	48	135	1423.5	1.2
**62.0**	18	90	200.0	0.3
**59.1**	18	90	266.7	0.3
**56.9**	18	90	322.2	0.3
**53.0**	18	90	433.3	0.3
**48.3**	18	90	588.9	0.3
**46.0**	18	90	677.8	0.3
**43.9**	18	90	766.7	0.3
**115.4**	24	120	28.5	0.9

Table S6 Summary of the five different literature reported conditions for which the successful synthesis of MOF-801 has been achieved in DMF using $ZrOCl_2 \cdot 8H_2O$ as the metal source and formic acid (FA) as the modulator.[8] The quantity of $ZrOCl_2 \cdot 8H_2O$ presented in the table have been scaled to a total volume of reaction (DMF + FA) equal to 10 mL.

$ZrOCl_2 \cdot H_2O$ (mg/10 mL)	Time (hr)	Temperature (°C)	FA:Zr	Zr:Linker
**57.1**	24	120	200.7	1.0
**592.6**	6	130	37.1	1.0
**592.6**	2	140	37.1	1.0
**592.6**	6	130	37.1	1.0
**592.6**	6	130	37.1	1.0

Batch 1 PXRD Data:

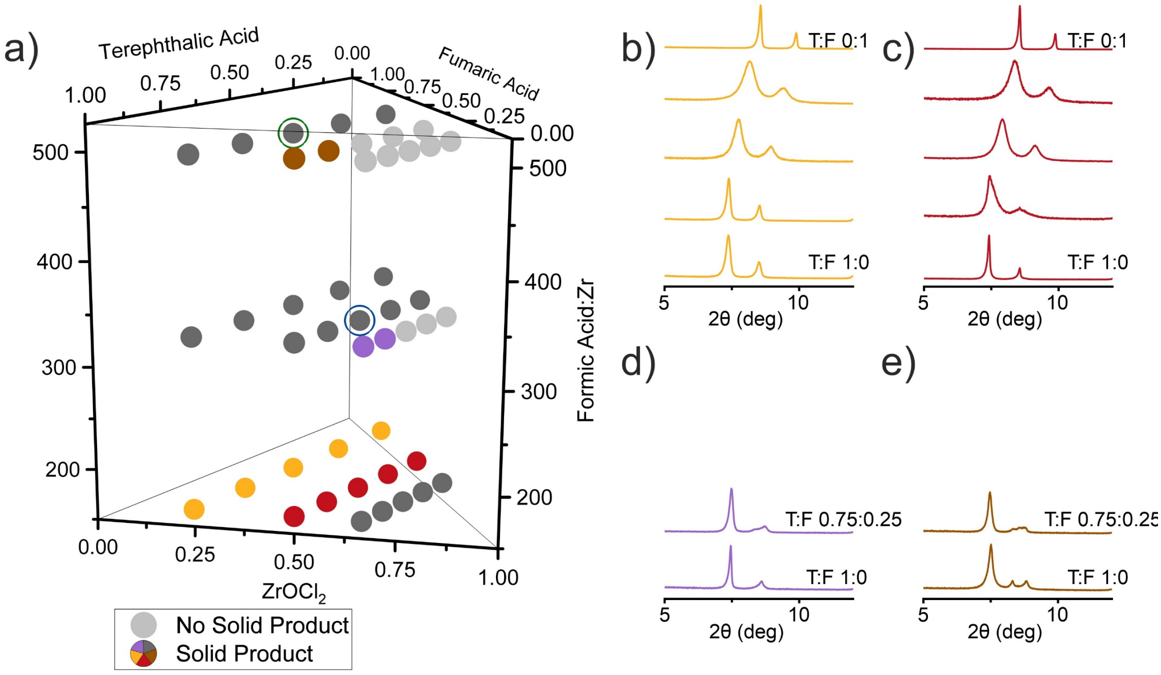

Figure S2 (a) Compositions of the 45 reaction mixtures selected for the initial batch synthesis to explore the system $ZrOCl_2$, terephthalic acid, fumaric acid and formic acid with DMF as the solvent. The light grey symbols identify reactions which lead to no solid product. The remaining symbols identify reactions which lead to a solid product, and are colour coded to correspond to the stack of PXRD patterns of the same colour in panels (b) – (e). PXRD patterns for the dark grey points are given in Figure 2 of the main text. The points circled in blue and green represent the major and minor hits respectively. (b) – (e) PXRD (Cu Kα_1, λ = 1.5406 Å) patterns for the remaining 14 samples, identified by the black points in Figure 2. In each stack of PXRD patterns, the only variable is the T:F ratio, with the bottom pattern corresponding to pure terephthalic acid and the top to pure fumaric acid.

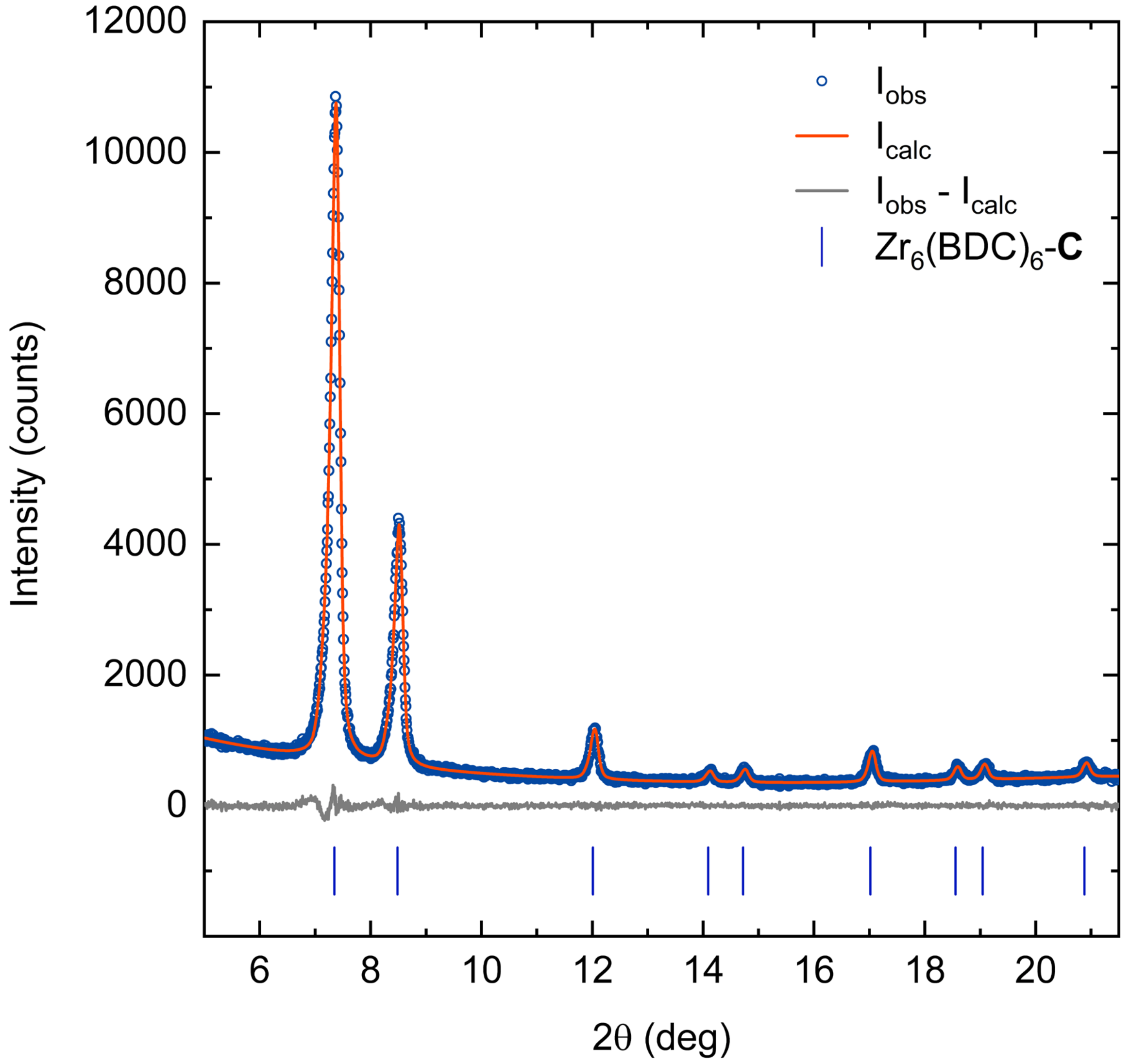

Figure S3 Result of the whole powder pattern refinement carried out with the Le Bail approach on the PXRD pattern of the cubic phase (**C**) material, $Zr_6(BDC)_6$-**C** (Cu Kα_1, λ = 1.5406 Å, blue symbols), synthesised in the reaction with composition Zr:T:F = 0.25:0.75:0 and FA:Zr = 167, calculated and difference traces (orange and grey, respectively; R_p = 0.031; R_{wp} = 0.041), starting from the known structure of UiO-66.[9] The positions of the Bragg reflections are indicated by blue ticks.

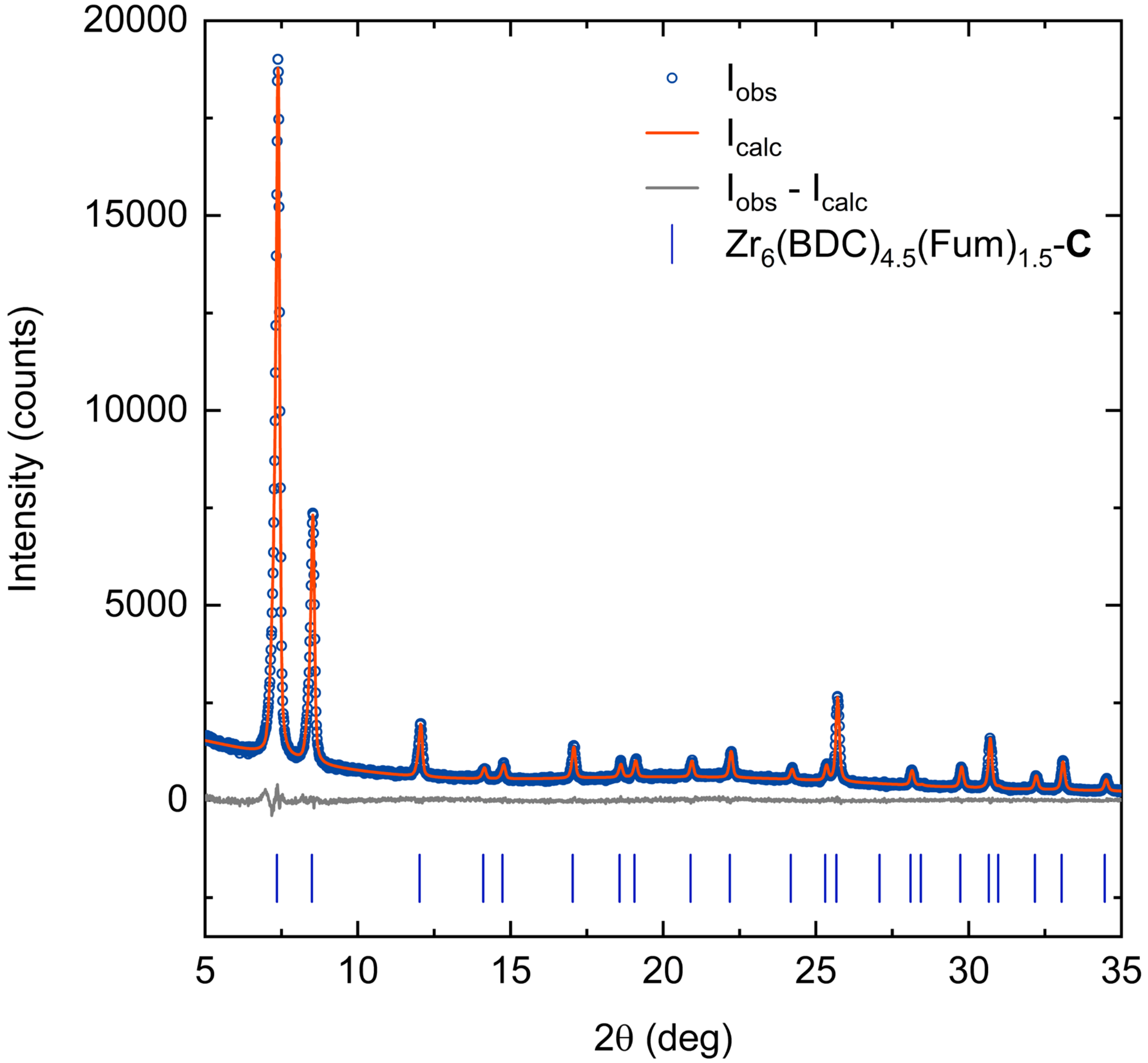

Figure S4 Result of the whole powder pattern refinement carried out with the Le Bail approach on the PXRD pattern of the cubic phase (**C**) material, $Zr_6(BDC)_{4.5}(Fum)_{1.5}$-**C** (Cu K$\alpha_1$, λ = 1.5406 Å, blue symbols), synthesised in the reaction with composition Zr:T:F = 0.25:0.56:0.19 and FA:Zr = 167, calculated and difference traces (orange and grey, respectively; R_p= 0.033; R_{wp}= 0.043), starting from the known structure of UiO-66.[9] The positions of the Bragg reflections are indicated by blue ticks.

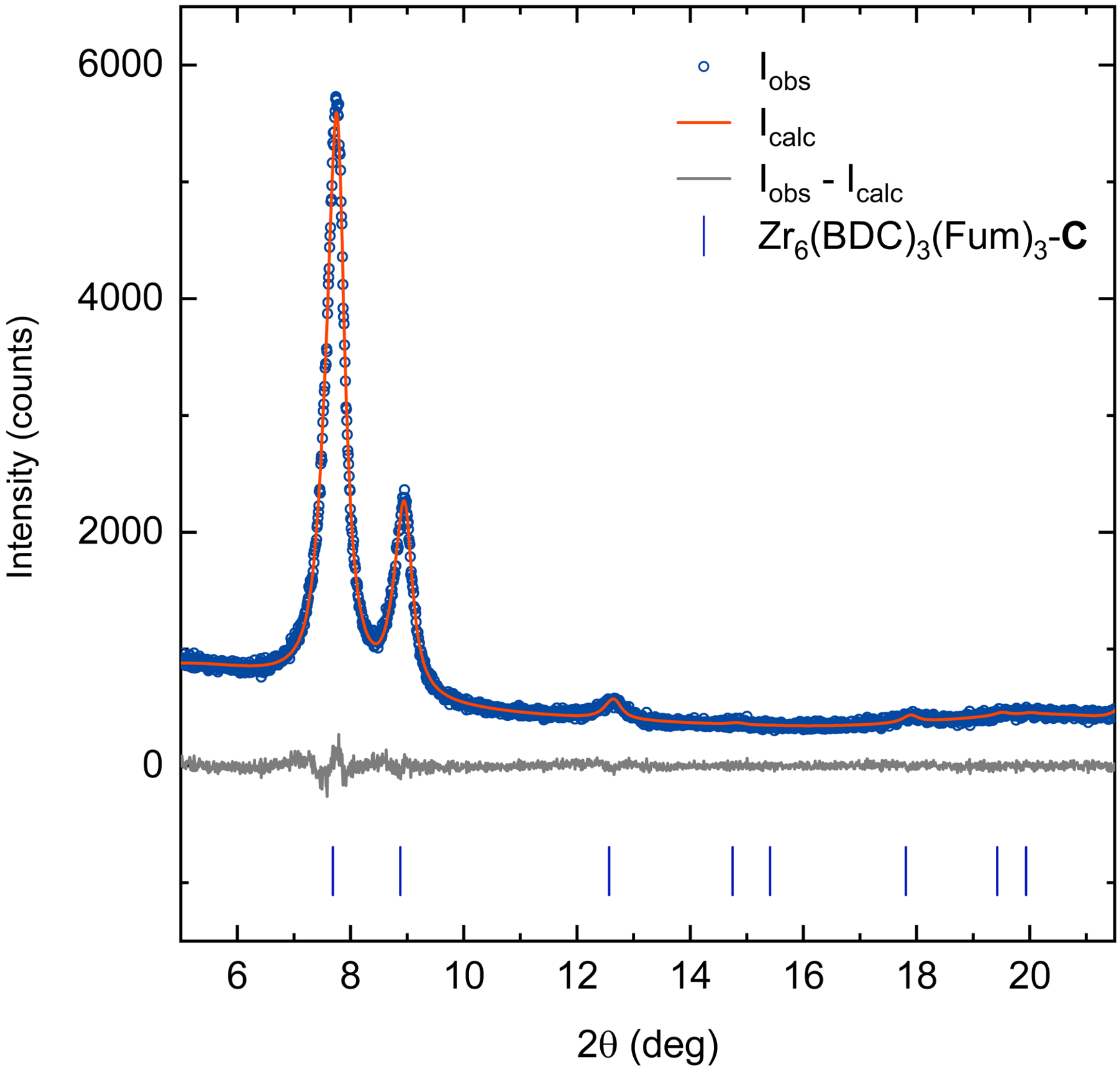

Figure S5 Result of the whole powder pattern refinement carried out with the Le Bail approach on the PXRD pattern of the cubic phase (**C**) material, $Zr_6(BDC)_3(Fum)_3$-**C** (Cu Kα_1, λ = 1.5406 Å, blue symbols), synthesised in the reaction with composition Zr:T:F = 0.25:0.375:0.375 and FA:Zr = 167, calculated and difference traces (orange and grey, respectively; R_p = 0.032; R_{wp} = 0.042), starting from the known structure of UiO-66.[9] The positions of the Bragg reflections are indicated by blue ticks.

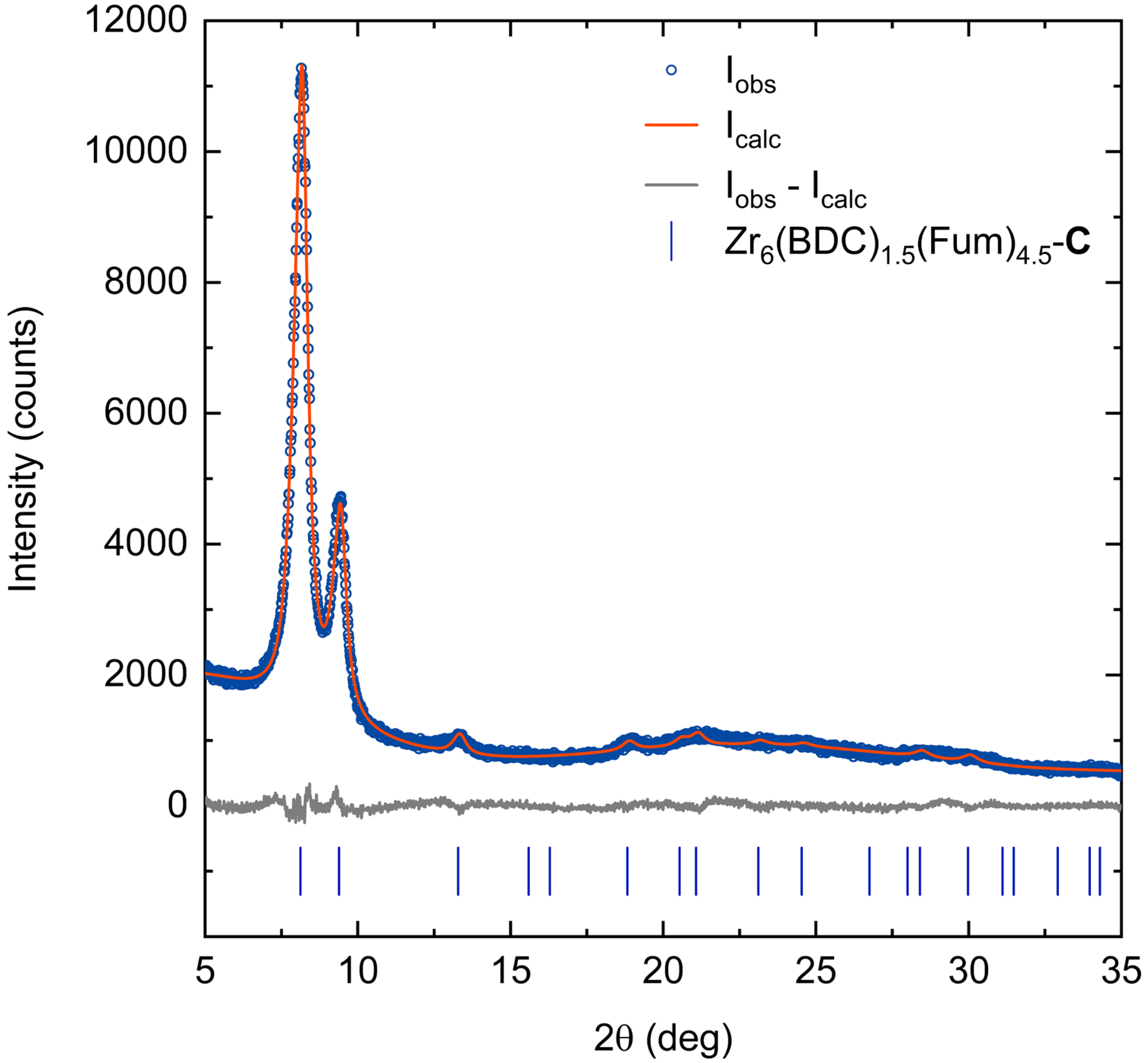

Figure S6 Result of the whole powder pattern refinement carried out with the Le Bail approach on the PXRD pattern of the cubic phase (**C**) material, $Zr_6(BDC)_{1.5}(Fum)_{4.5}$-**C** (Cu K$\alpha_1$, λ = 1.5406 Å, blue symbols), synthesised in the reaction with composition Zr:T:F = 0.25:0.19:0.56 and FA:Zr = 167, calculated and difference traces (orange and grey, respectively; R_p = 0.030; R_{wp} = 0.040), starting from the known structure of UiO-66.[9] The positions of the Bragg reflections are indicated by blue ticks.

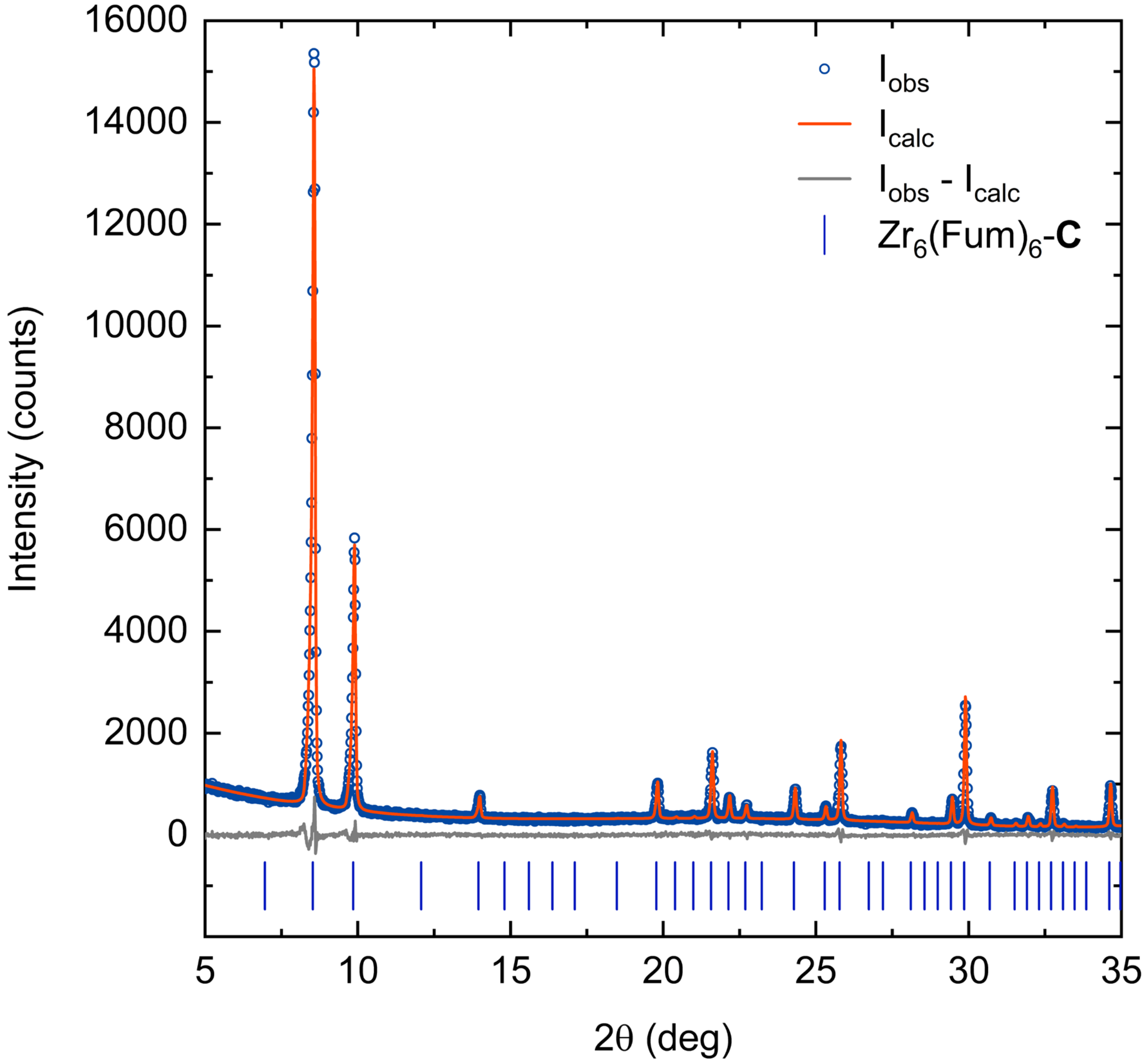

Figure S7 Result of the whole powder pattern refinement carried out with the Le Bail approach on the PXRD pattern of the cubic phase (**C**) material, $Zr_6(Fum)_6$-**C** (Cu Kα_1, λ = 1.5406 Å, blue symbols), synthesised in the reaction with composition Zr:T:F = 0.25:0:0.75 and FA:Zr = 167, calculated and difference traces (orange and grey, respectively; R_p = 0.041; R_{wp} = 0.055), starting from the known structure of MOF-801.[10] The positions of the Bragg reflections are indicated by blue ticks.

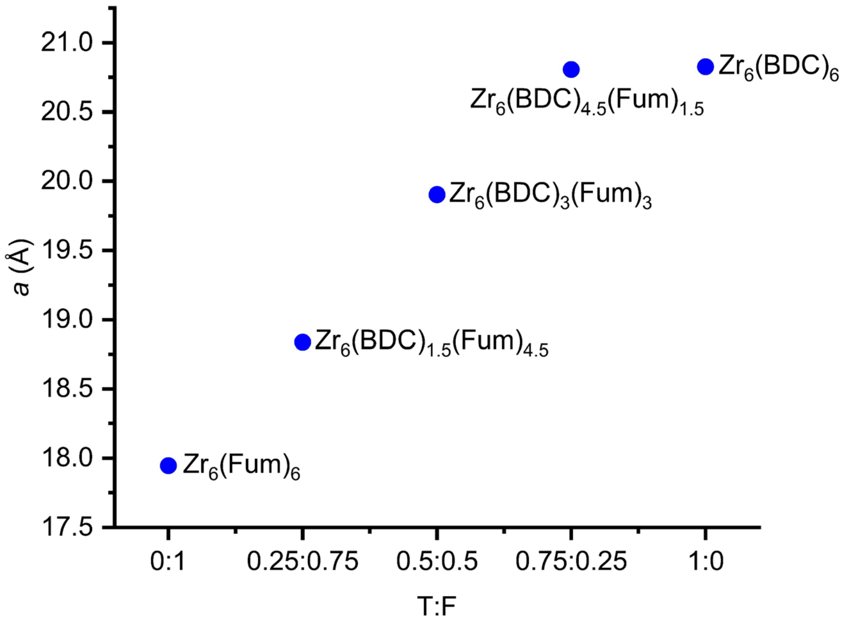

Figure S8 Variation of the unit cell parameter *a* of the five cubic phase materials (Figures S3 – S7) synthesised in the first batch with composition Zr:(T+F) = 0.25:0.75 and FA:Zr = 167, which vary only by their T:F molar ratio.

Table S7 The values of the unit cell parameter *a* (Figure S8) retrieved from the whole powder pattern refinements on these five cubic phase compounds (Figures S3 – S7), carried out with the Le Bail approach.

	a **(Å)**
**$Zr_6(Fum)_6$-C**	17.9457(2)
**$Zr_6(BDC)_{1.5}(Fum)_{4.5}$-C**	18.837(11)
**$Zr_6(BDC)_3(Fum)_3$-C**	19.903(13)
**$Zr_6(BDC)_{4.5}(Fum)_{1.5}$-C**	20.8067(5)
**$Zr_6(BDC)_6$-C**	20.827(2)

Supplementary Note 1:
Calculation of T:F Ratio by ^{1}H NMR:

The incorporation of both the terephthalate and fumarate linkers in the materials was determined by ^{1}H NMR of the digested samples in a mixture of NaOD (60 μL) and D_2O (640 μL). The relative quantities in which the two linkers present in the samples was determined from the relative integrations of the corresponding peaks for each of these species.

In each spectrum the relative integration of terephthalate, which has 4 protons, was taken to be equal to 1. Therefore, as each molecule of fumarate contains only 2 protons, half that of terephthalate, in order to calculate the quantity of formate present and the T:F ratio of each sample, the relative integration of the fumarate peak in the spectra was doubled, an equivalent operation to dividing the value of the integration by the number of protons of fumarate, 2, and multiplying by the number of protons of terephthalate, 4, which the integrations were calculated relative to.

Batch 1 ^{1}H NMR Data:

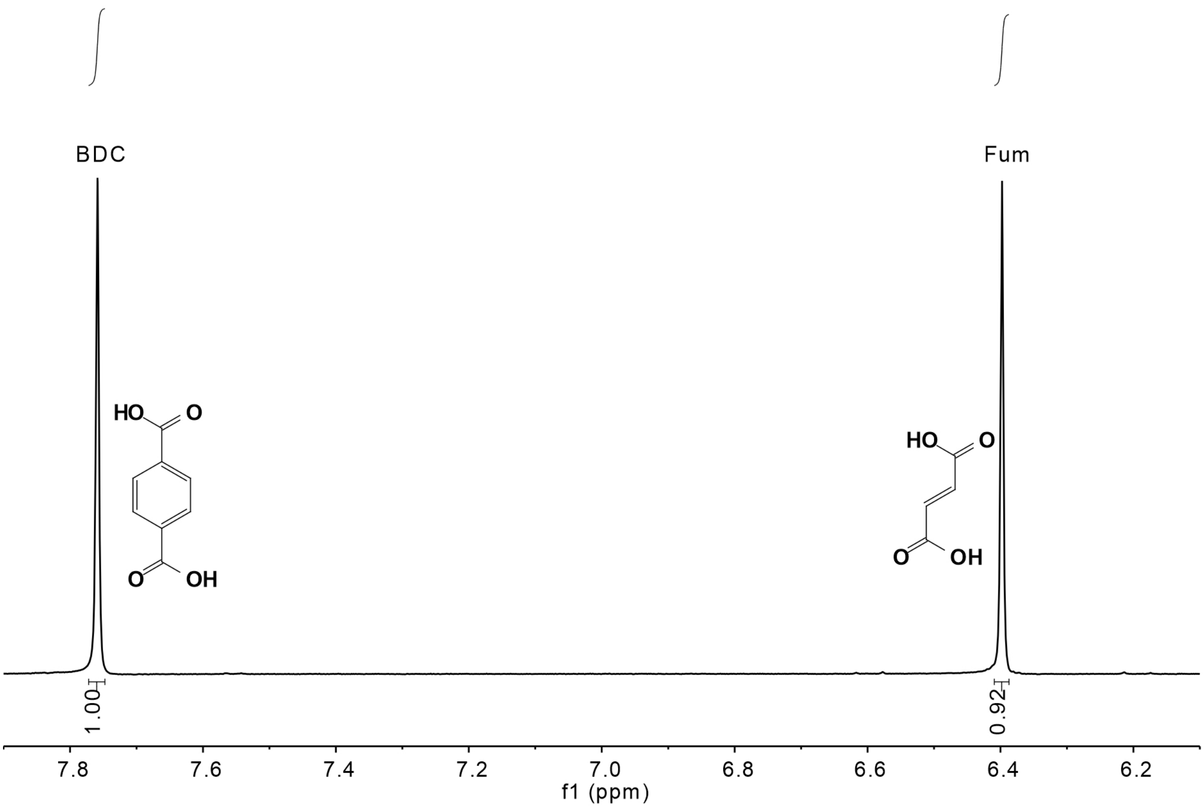

Figure S9 ^{1}H NMR spectrum of the cubic phase material with composition Zr:T:F = 0.25:0.19:0.56 and FA:Zr = 167, synthesised in the first batch synthesis, after digestion in a mixture of NaOD (60 µL) and D_2O (640 µL). From the relative integrations of the peaks corresponding to terephthalate and fumarate, the molar ratio in which the two linkers are present in the material was calculated to be T:F = 1:1.84. This is further evidenced by the PXRD pattern of the material (Figure S6) which confirms that the material exhibits a cubic phase in which the unit cell parameter *a* (*a* = 18.837(11) Å, Figure S8) is larger than that of MOF-801 (*a* = 17.9457(2) Å, Figure S8).

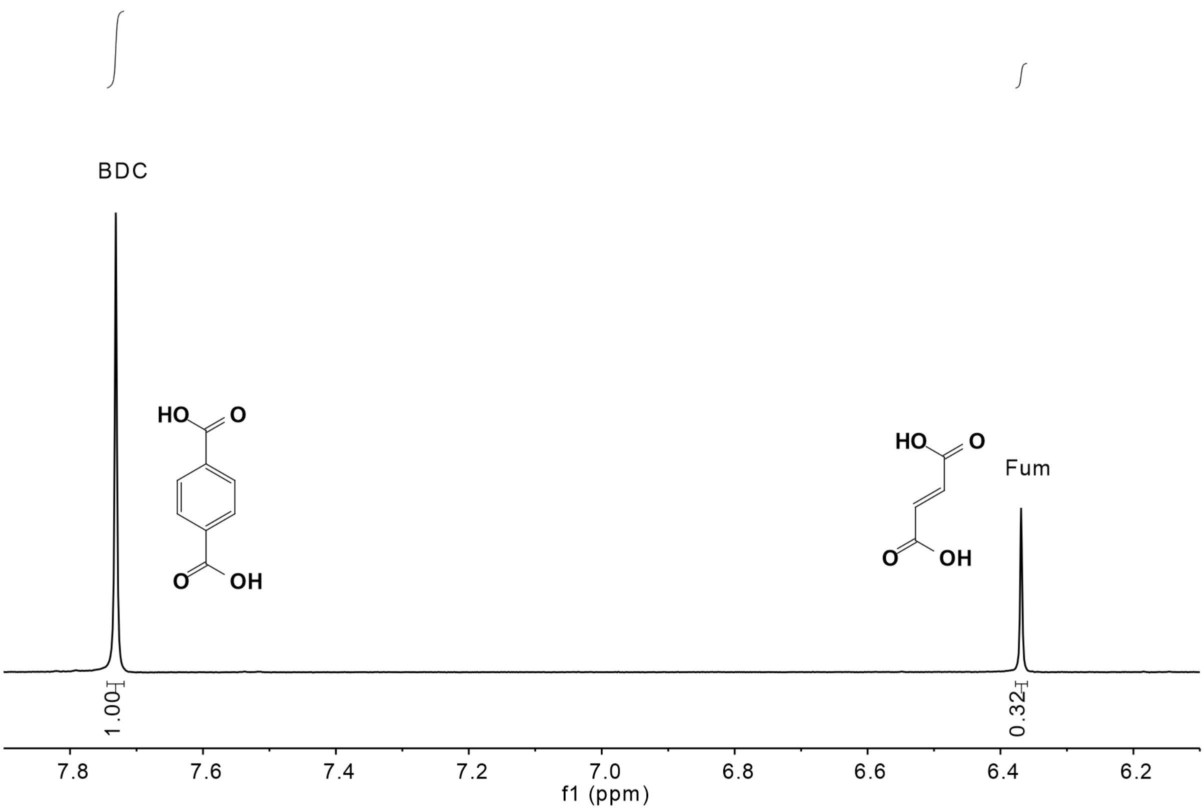

Figure S10 ^{1}H NMR spectrum of the cubic phase material with composition Zr:T:F = 0.5:0.25:0.25 and FA:Zr = 167, synthesised in the first batch synthesis, after digestion in a mixture of NaOD (60 µL) and D_2O (640 µL). The nominal composition of the material from the reaction composition has a T:F molar ratio of T:F = 1:1, however from the relative integrations of the peaks corresponding to terephthalate and fumarate, the molar ratio in which the two linkers are present in the material was calculated to be T:F = 1:0.64. This therefore suggests that under these reaction conditions, the system shows a preference for terephthalate over fumarate.

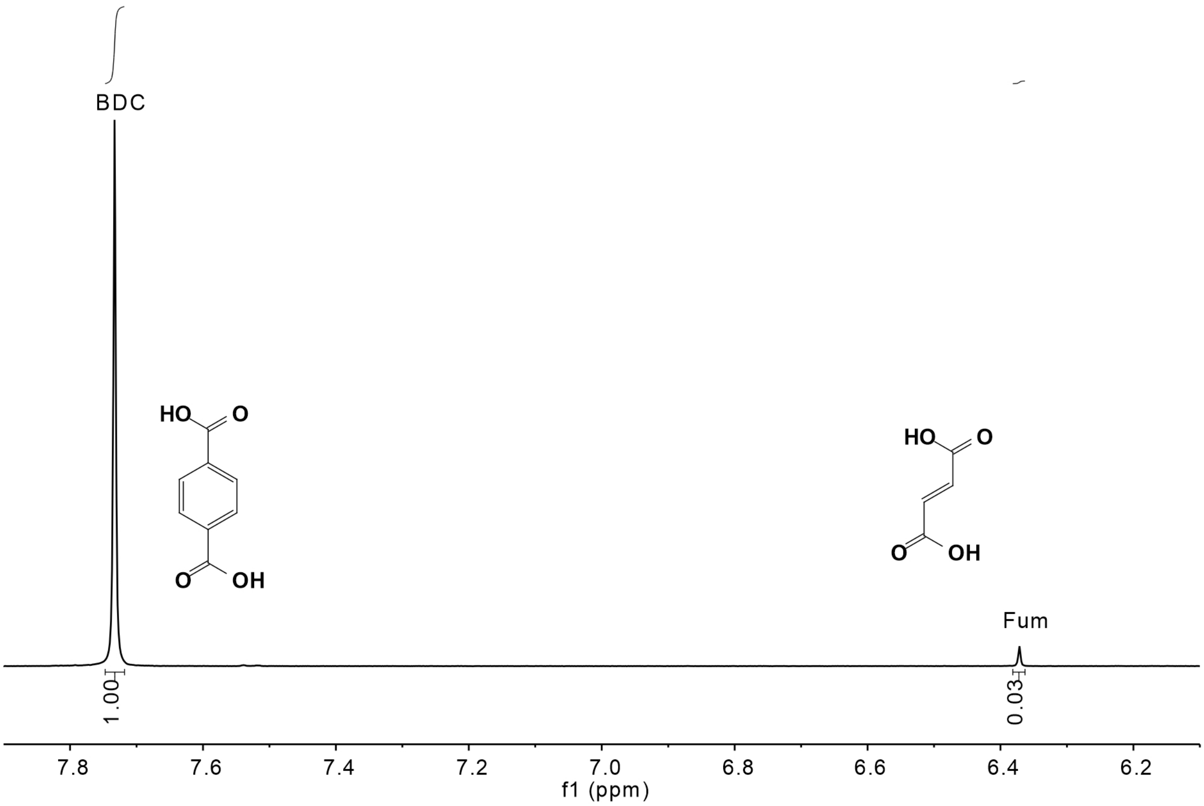

Figure S11 ^{1}H NMR spectrum of the cubic phase material with composition Zr:T:F = 0.667:0.25:0.083 and FA:Zr = 167, synthesised in the first batch synthesis, after digestion in a mixture of NaOD (60 µL) and D_2O (640 µL). From the relative integrations of the peaks corresponding to terephthalate and fumarate, the molar ratio in which the two linkers are present in the material was calculated to be T:F = 1:0.06. The incorporation of fumarate into the material is extremely low, significantly lower than the ratio in the nominal composition of the material, where the molar T:F ratio was T:F = 3:1. This is further evidenced by the PXRD pattern of the material (Figure 3e) which shows the cubic phase of UiO-66.

Table S8 Summary of the experimental molar T:F ratios for the three cubic phase materials, synthesised in the first batch synthesis, for which ^{1}H NMR data was obtained (Figures S9 – S11). The experimental T:F molar ratios were calculated from the relative integrations of the terephthalate and fumarate peaks in the spectra (Figures S9 – S11) and the nominal T:F molar ratios are determined by the composition of the reaction mixture.

Zr:T:F	FA:Zr	Nominal T:F (Molar)	Experimental T:F (Molar)
0.25:0.19:0.56	167	1:3	1:1.84
0.5:0.25:0.25	167	1:1	1:0.64
0.667:25:0.083	167	3:1	1:0.06

Supplementary Note 2:

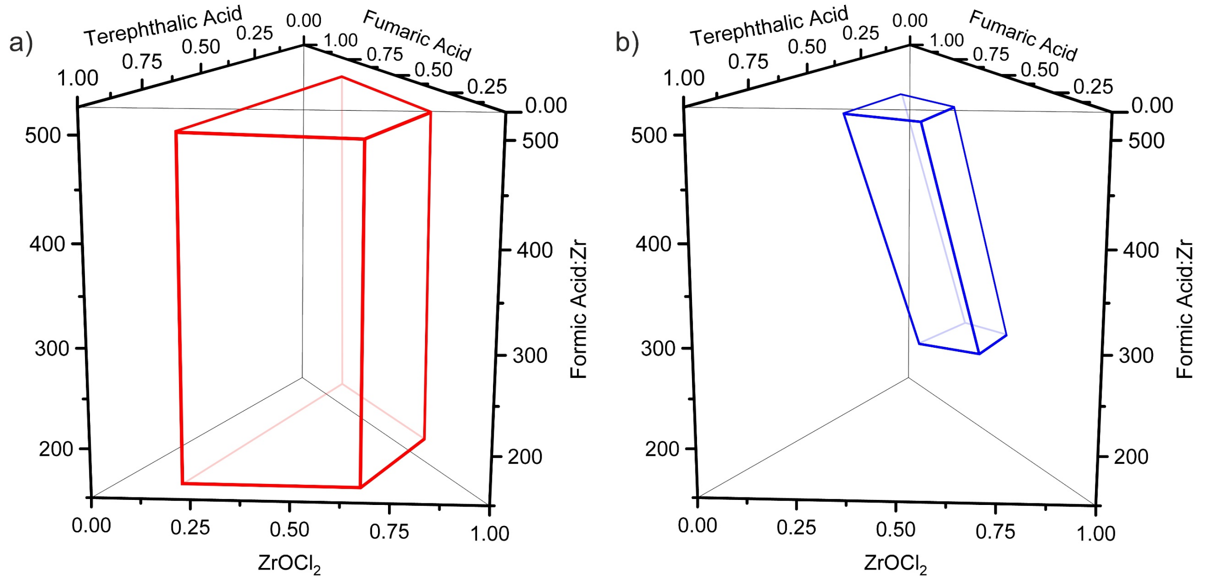

Figure S12 The volume of the chemical space explored in (a) the first iteration of the synthesis (highlighted by the red trapezoid), which was covered evenly by a set of 45 points (Table S2), and (b) the smaller region of chemical space explored in the second iteration of the synthesis (highlighted by the blue trapezoid), which was densely covered by 54 points (Table S4) and selected to focus on the major and minor hits obtained in batch 1. The volume of the space explored in the second iteration of the synthesis was approximately 15 times smaller than that which was explored in the first (Supplementary Note 2).

Calculation of the Volume of Chemical Space Explored:

The region of the chemical space explored in the first iteration of the synthesis (Figure S12a) can be represented by a trapezoid which is defined by the 45 points prepared in the first batch of syntheses (Table S2). The area of the trapezium which defines the base of the trapezoid was calculated to be 0.197, where the parallel edges of the shape have lengths of 0.750 and 0.333 and the distance between them is calculated to be 0.363. The height of the trapezoid was calculated as the difference in FA:Zr ratio between the points which define the top (FA:Zr = 501) and bottom (FA:Zr = 167) faces of the trapezoid and was found to be 334. Using these dimensions, the volume of the space explored in the first iteration was calculated to be 65.80. The volume of the convex polyhedron (Figure S12b), which represents the region of chemical space covered by the 54 points of the second batch synthesis (Table S4), is calculated by its convex hull using the Qhull library as provided in SciPy.[11] The convex hull is represented by eight points, each one described by three coordinates which correspond to the Zr and F values of the composition (Zr:T:F) and FA:Zr ratio of the reaction mixture represented by the point. The Qhull algorithm tries to identify the smaller convex set that contains the points, which corresponds to a volume of 4.49 units. The python code used for the volume calculation is provided below:

```
import numpy as np
from scipy.spatial import ConvexHull

points = np.array([[0.5, 0.3125, 292.43847],
[0.5, 0.1875, 292.43847],
[0.62963, 0.23148, 292.43847],
[0.62963, 0.13889, 292.43847],
[0.24812, 0.46992, 501.3231],
[0.24812, 0.28195, 501.3231],
[0.42857, 0.35714, 501.3231],
[0.42857, 0.21429,    501.3231]], dtype=float)

hull=ConvexHull(points)
print(hull.volume)
>> 4.4902350056624165
```

From the calculated volumes of the polygons which represent the regions of chemical space explored in batches 1 and 2, 65.80 and 4.49 respectively, the volume of the region defined by the points of batch 2 was calculated to be 14.7 times smaller than that which is defined by the points of batch 1.

Batch 2 PXRD Data:

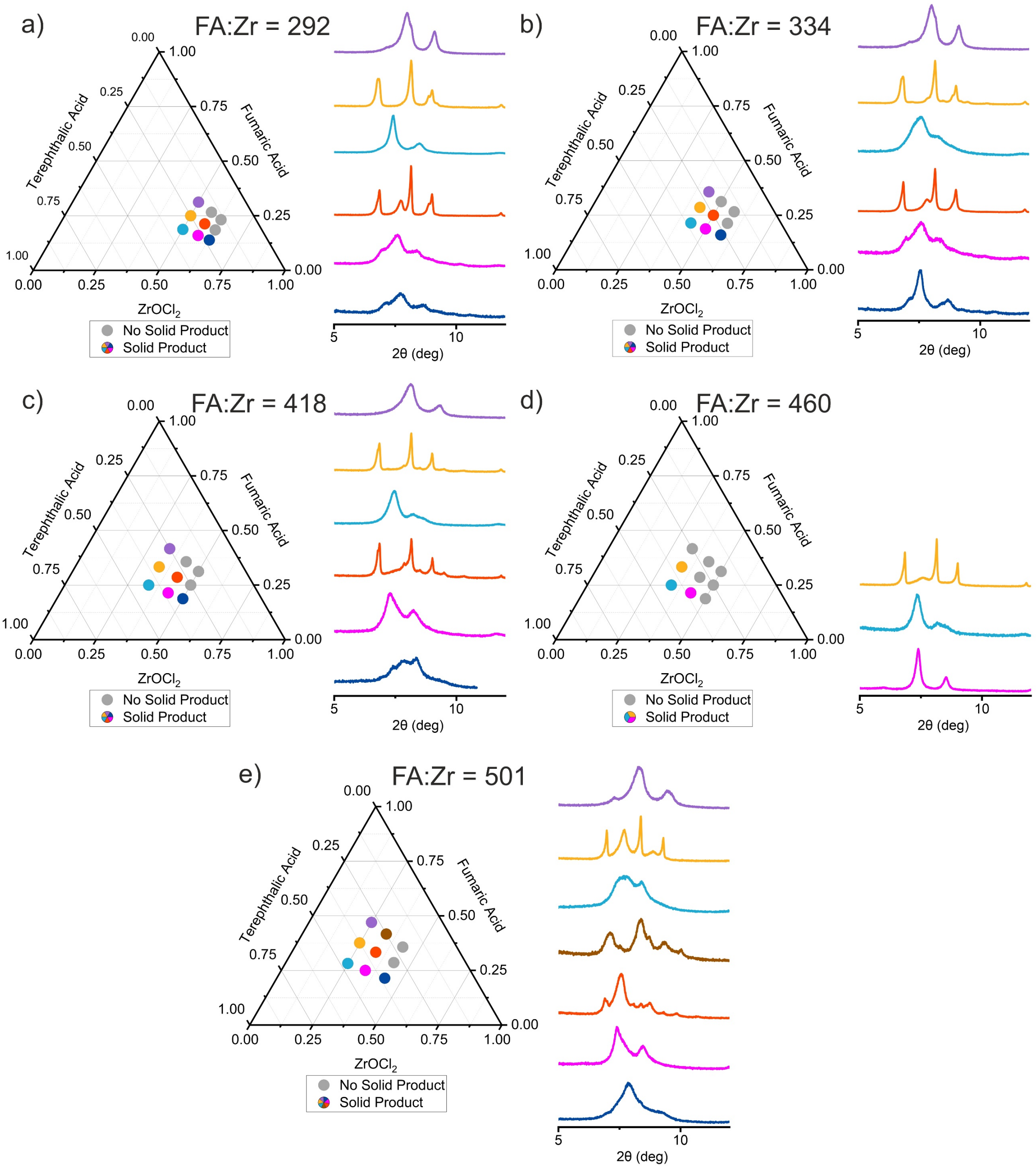

Figure S13 PXRD (Cu Kα_1, λ = 1.5406 Å) patterns of the 28 samples in the second batch of syntheses that are obtained from reactions with FA:Zr molar ratios other than FA:Zr = 376 (PXRD patterns for the 4 samples obtained from reactions with FA:Zr = 376 are shown in Figure 4). In each panel (a) – (e) the FA:Zr molar ratio used is quoted, with the points in the ternary plot representing the compositions of the 9 reactions prepared at that FA:Zr ratio. Points identifying reactions which yielded a solid product are colour coded, with each point corresponding to the PXRD pattern of the same colour in the PXRD pattern stack to the right of the ternary plot. Reactions which did not yield a solid product are identified by grey points.

Summary of All Reaction Compositions:

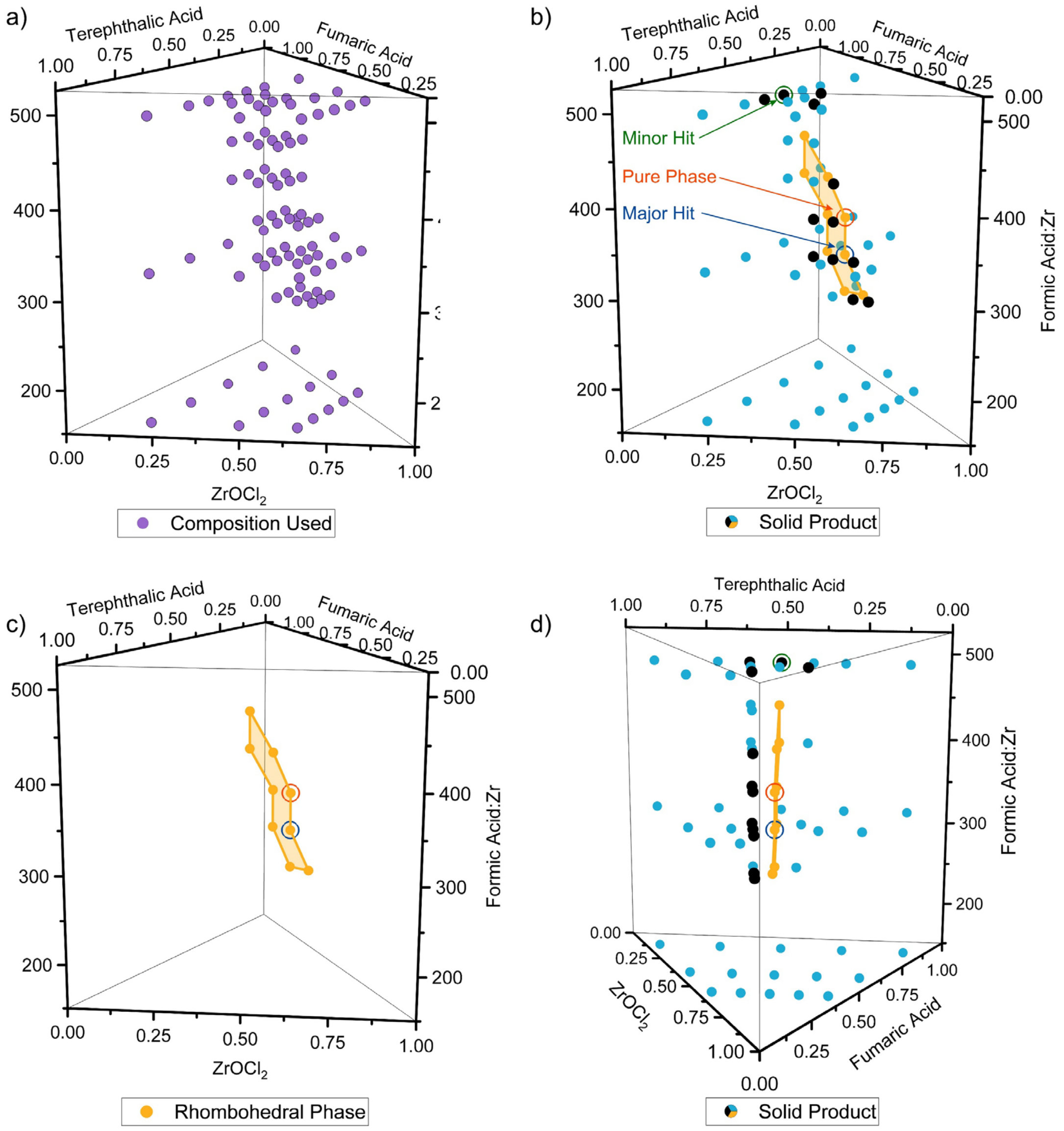

Figure S14 (a) Compositions of the 97 different reaction (purple symbols) performed in the two batches for the exploration of the system $ZrOCl_2$ - terephthalic acid - fumaric acid - formic acid with DMF as solvent. A total of 99 reactions were prepared across the two batches (45 in the first batch and 54 in the second batch). The two points identified as the major and minor hits in batch 1 (circled blue and green points respectively in (b)) were included in the design of batch 2. In (b) these 97 unique compositions are colour coded to indicate their outcome. Out of the 97 different reaction compositions evaluated, only the 9 reactions indicated by the yellow symbols led to any formation of the rhombohedral phase of $Zr_6(BDC)_3(Fum)_3$, with the remaining 55 reactions yielding solids, which exhibit cubic phases (light blue points), or a complex mixture of multiple phases (black points) which cannot be easily identified when characterised by PXRD. In (c), for clarity, only the 9 points which yield the rhombohedral phase are shown, and the narrow region of the explored chemical space in which this phase was obtained indicated by the yellow shaded area. From the view of the chemical space in (d), where only the reaction compositions which led to the formation of a cubic phase (light blue points) or the rhombohedral phase (yellow points), it is clear that in comparison to the disordered mixed-linker MOFs with cubic structures, which form over a broad range of the

explored chemical space, the ordered rhombohedral structure of $Zr_6(BDC)_3(Fum)_3$ forms over a considerably narrower range of compositions, all of which require the use of equal quantities of terephthalate and fumarate (T:F = 1:1). Even within this highly restricted region, there is an even narrower range where the major hit and pure phase points lie, and thus where the new linker-ordered phase can be obtained with high purity. This indicates that forming an ordered mixed-linker MOF requires a highly specific set of compositions with little tolerance for change in any one of the parameters. This emphasises the importance of adopting a high-throughput (HT) approach in order to densely cover the chemical space explored and thus locate the conditions required for single-step self-assembly of ordered multiple linker MOFs.

$Zr_6(BDC)_3(Fum)_3$ PXRD Indexing and Rietveld Refinement:

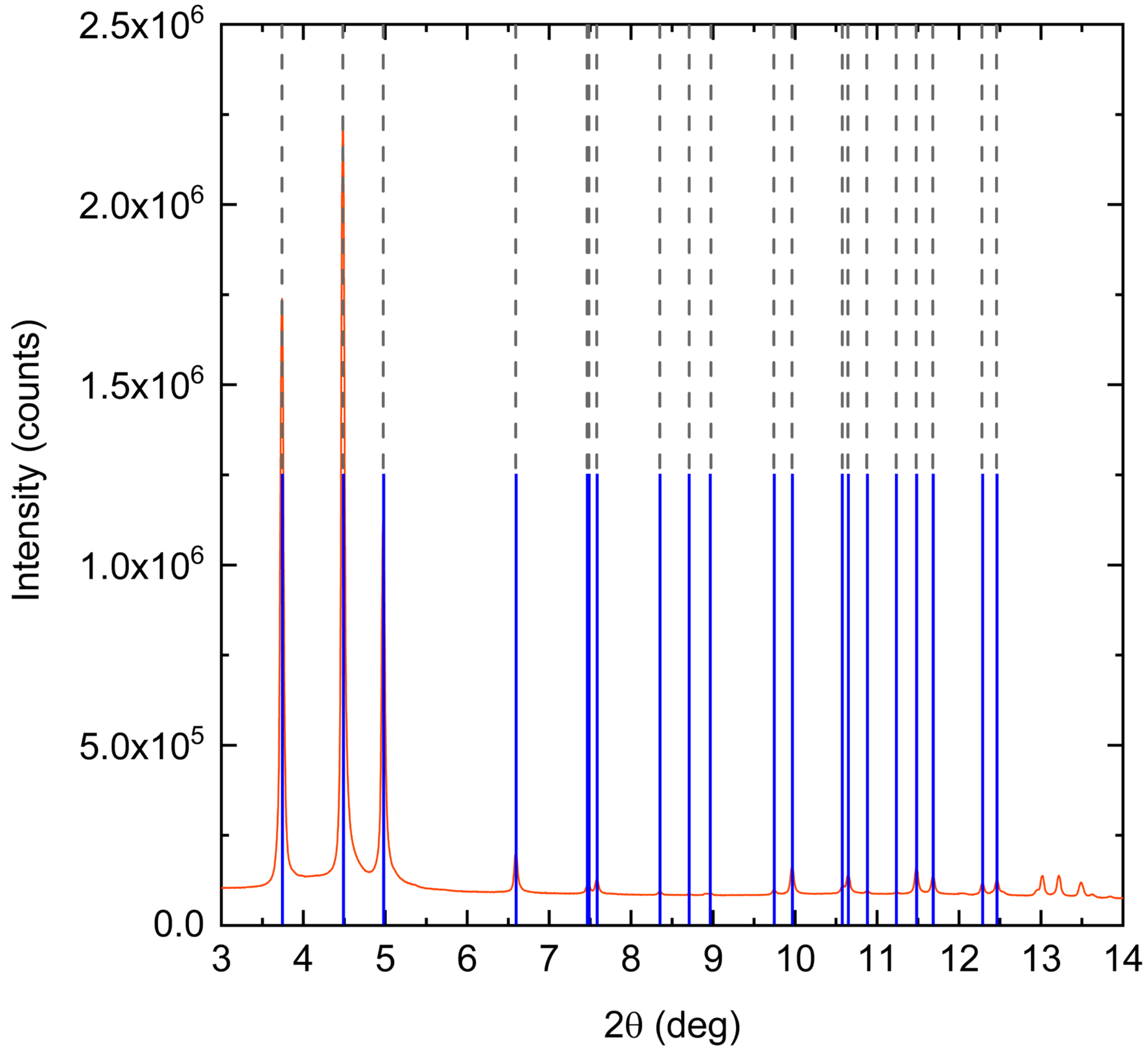

Figure S15 Graphic output of the indexing procedure carried out on $Zr_6(BDC)_3(Fum)_3$ (I11, λ = 0.826596(10) Å) using a standard peak search followed by profile fitting to estimate the peak positions and providing approximate unit cell parameters through the Singular Value Decomposition algorithm[1] as implemented in TOPAS-Academic V5[2]. Space Group: $R\bar{3}$ (space group no. 148), Cell parameters: $a = b$ = 12.7031 Å, c = 38.0030 Å, vol = 5310.911 $Å^3$, GOF = 41.52. Colour code: observed PXRD pattern, orange line; observed peak position, vertical blue lines; calculated peak positions, vertical dashed grey lines.

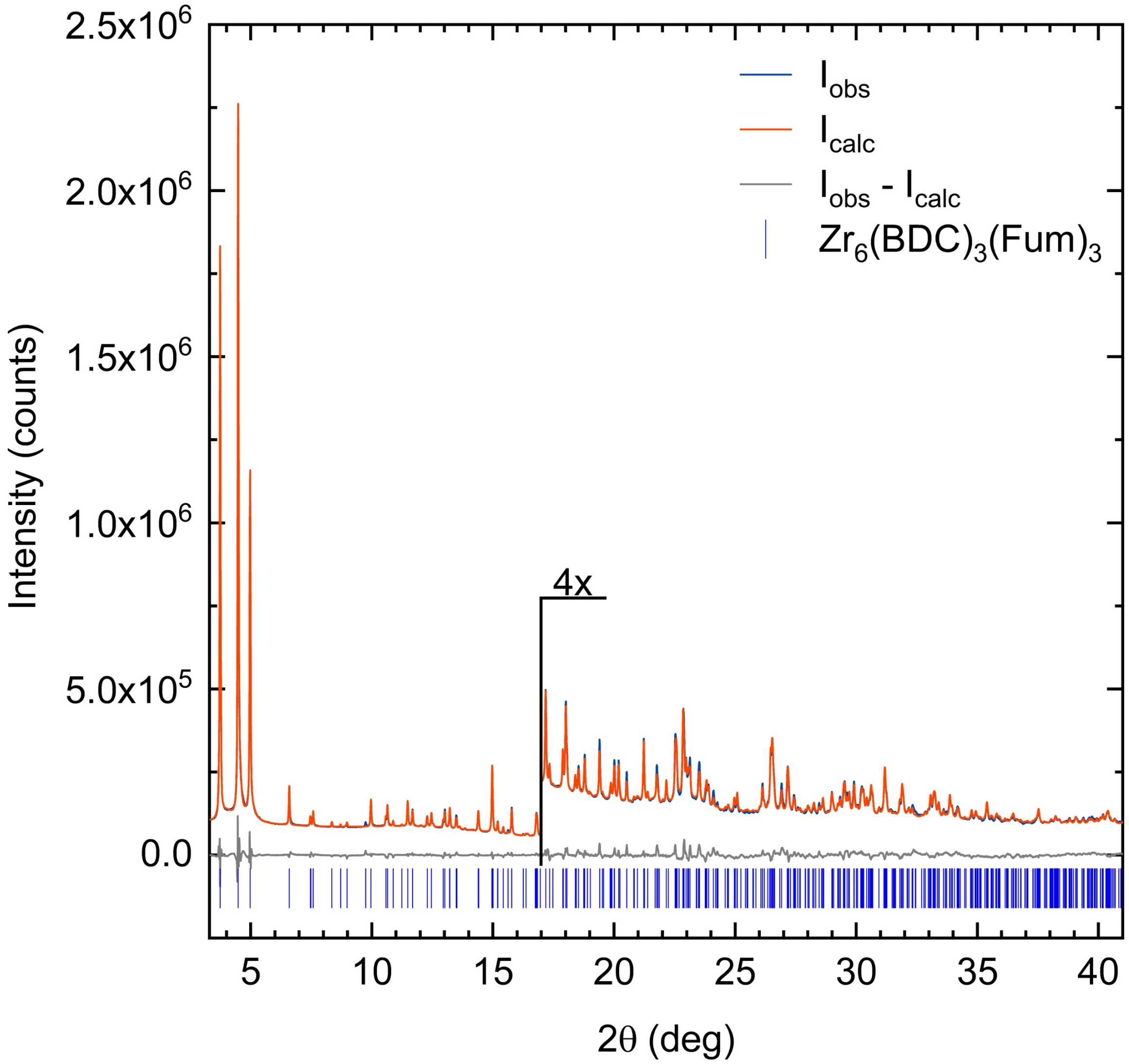

Figure S16 Graphical result of the final Rietveld refinement carried out on $Zr_6(BDC)_3(Fum)_3$ (I11, λ = 0.826596(10) Å) with experimental, calculated and difference traces (blue, orange and grey, respectively). The blue ticks at the bottom indicate the positions of the Bragg reflections. Horizontal axis, 2θ (deg); vertical axis, intensity (counts). The vertical axis in the portion above ~17° has been magnified (4×) for clarity.

Crystallographic Data Table:

Table S9 Structure refinement against powder diffraction data of $Zr_6(BDC)_3(Fum)_3$.

	$Zr_6(BDC)_3(Fum)_3$
**Empirical Formula**	$Zr_6C_{46.48}H_{70.18}O_{44.76}$
**Formula Weight (g mol^{-1})**	1892.44
**Space Group**	$R\bar{3}$ (n. 148)
**Z**	3
**Density (g cm^{-3})**	1.77868
**Temperature (K)**	298.15
**Wavelength (Å)**	0.826596 (10)
**d – spacing range (Å)**	0.97813 – 12.65787
**Number of reflections**	1320
**Number of refined parameters**	49
**a (Å)**	12.69646(7)
**b (Å)**	12.69646(7)
**c (Å)**	37.9733(4)
**Volume (Å^3)**	5301.20(8)
**R_p**	2.26
**R_{wp}**	3.19
**R_{exp}**	0.41
**χ^2**	7.86
**CCDC**	2089846

Crystal Structure of $Zr_6(BDC)_3(Fum)_3$:

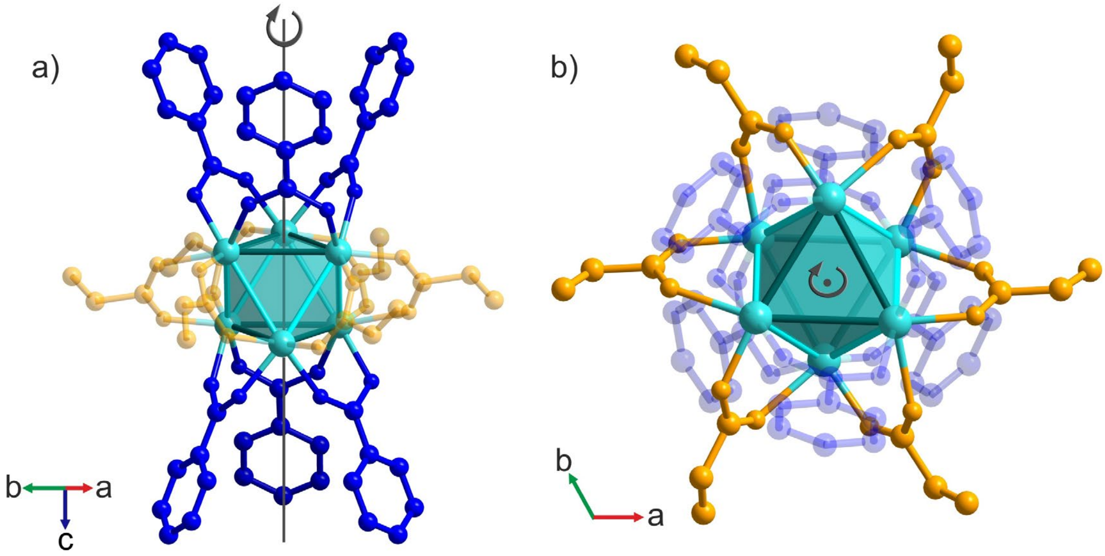

Figure S17 (a) The terephthalate linkers (blue) occupy the edges of the two equilateral triangular faces of the Zr_6 trigonal antiprism and are aligned with the threefold axis of the rhombohedral structure. (b) The remaining 6 edges of the antiprism are occupied by fumarate linkers (orange) which connect the equilateral triangular faces. The terephthalate-bridged equilateral triangular faces of the Zr_6 trigonal antiprism are rendered in a darker colour for clarity and all 12 bound linkers are shown in both (a) and (b).

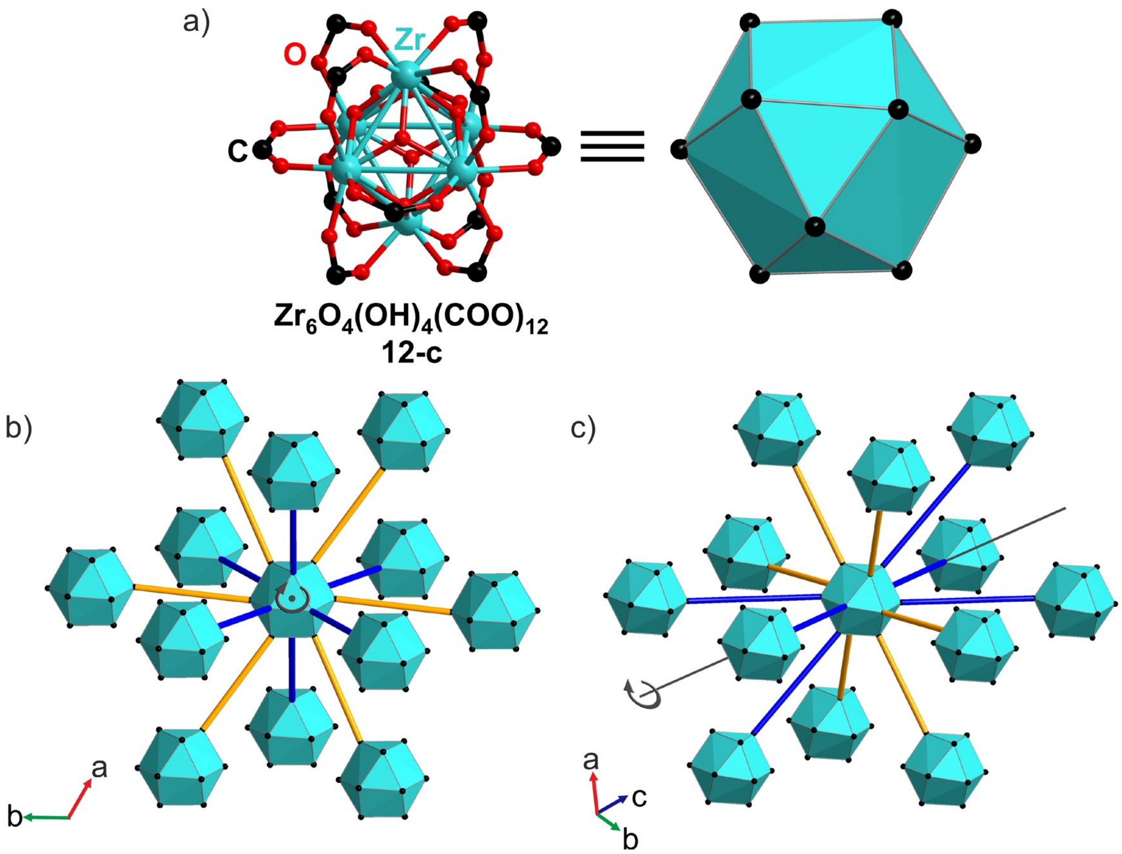

Figure S18 (a) $Zr_6O_4(OH)_4(COO)_{12}$ is represented as a distorted cuboctahedron, in which the vertices are the C atom (black) of the 12 carboxylate groups. The fcc packing arrangement of the distorted cuboctahedra (b) as viewed along the unique threefold axis of the rhombohedral structure and (c) oriented to view the structure along one of the other threefold axes which would be present in a cubic structure. Terephthalate linkers are represented by the long blue edges and fumarates by the short yellow edges. In both views each cuboctahedron is connected to six others by two fumarates and one terephthalate above and below, as well as to six other cuboctahedra in the same plane by four terephthalates and two fumarates which are arranged opposite one another.

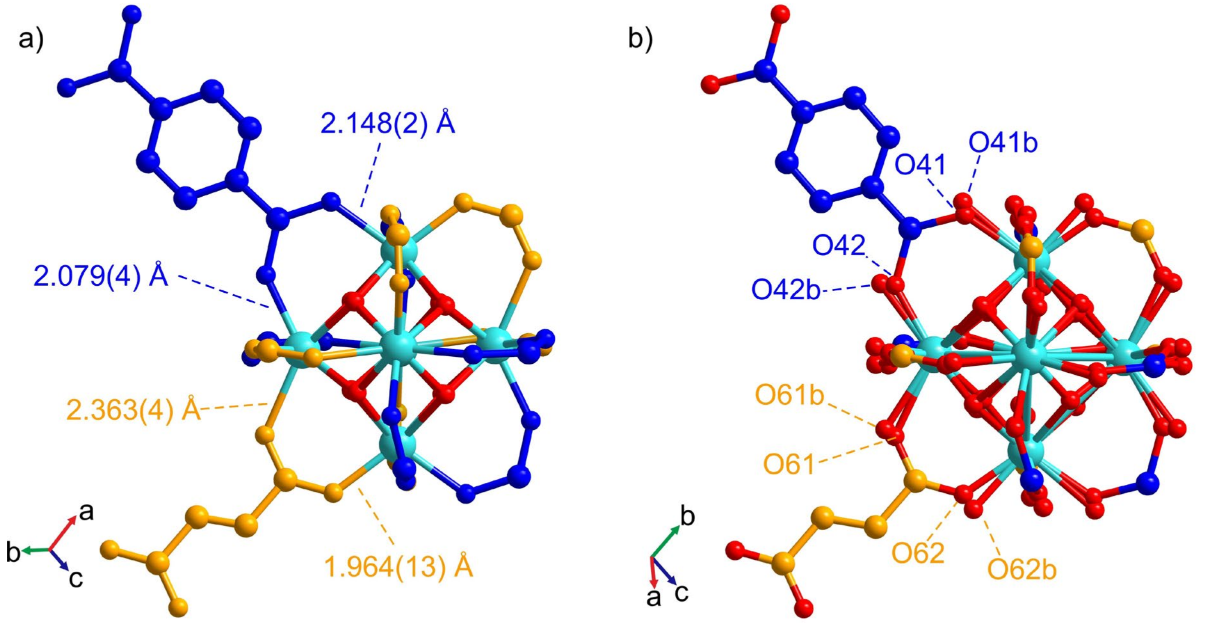

Figure S19 (a) Binding modes of terephthalate (blue) and fumarate (orange) linkers to the Zr_6 core. The zigzag shape of fumarate induces high asymmetry on the binding of its carboxylate group to the Zr_6 core, which is reflected in the difference in the $Zr\text{-}O_{Fumarate}$ bond lengths of 1.964(13) and 2.363(4) Å. The same level of asymmetry is not seen for the terephthalates, which bind in a more symmetrical mode to the Zr_6 core, with $Zr\text{-}O_{Terephthalate}$ bond lengths of 2.148(2) and 2.079(4) Å. (b) The additional oxygen sites O41b, O42b, O61b and O62b are associated with the structure defects, missing terephthalate and fumarate linkers, which have been modelled as formates. Similar sites have been observed in the UiO-66 structure.[5c, 6]

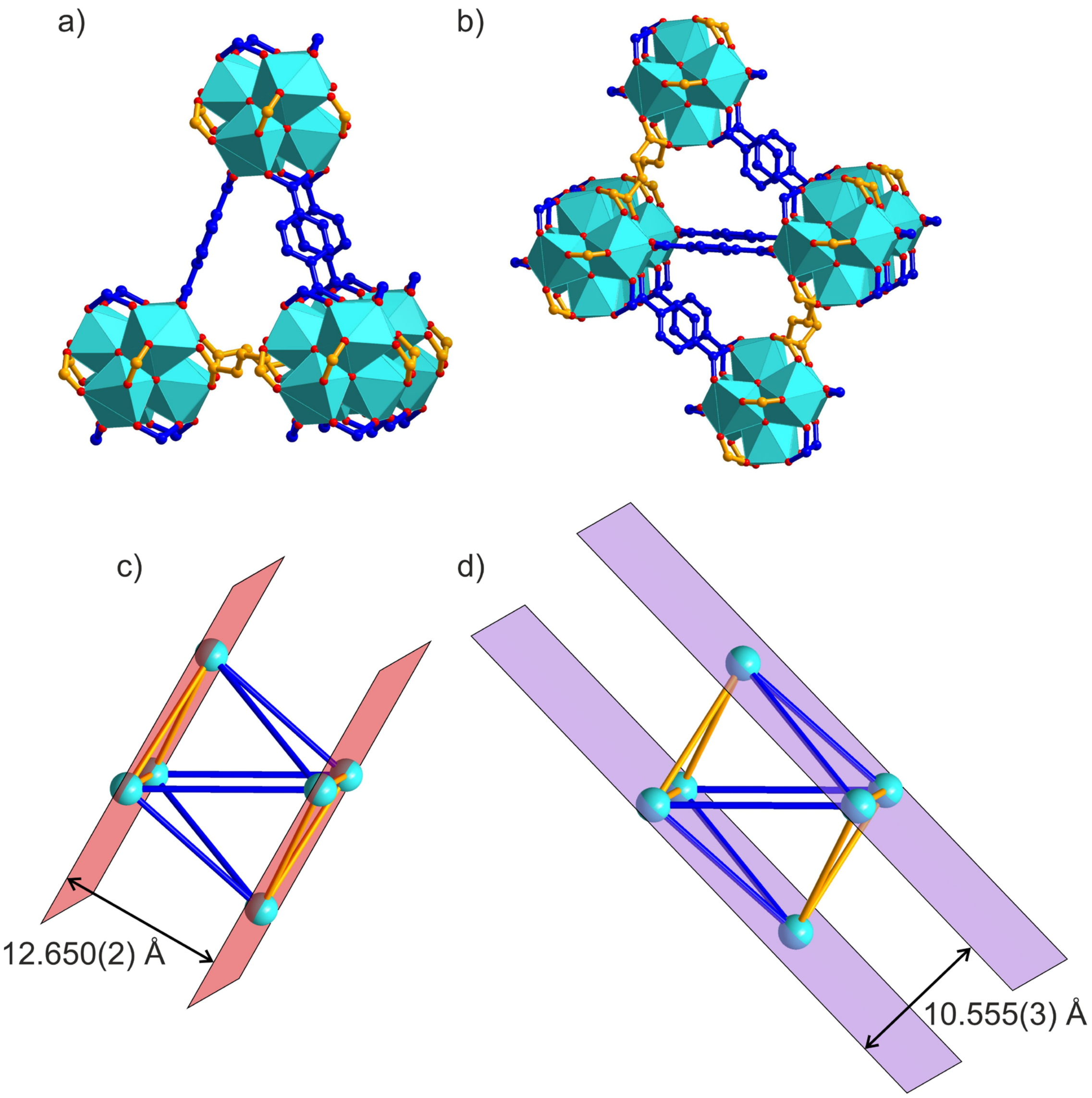

Figure S20 (a) The distorted tetrahedral, trigonal pyramidal, and (b) octahedral, trigonal antiprismatic, cages of $Zr_6(BDC)_3(Fum)_3$. These two cages are connected by two types of triangular windows. One of these triangular windows is fully composed of fumarates, 3F, and therefore adopts an equilateral triangular shape. The other triangular window is isosceles, with sides composed of two terephthalates and one fumarate, 2T1F. Fumarate in orange, terephthalate in blue. The degree of rhombohedral distortion in $Zr_6(BDC)_3(Fum)_3$ is expressed by the different distances between opposing windows of the same type measured through the centre of the octahedral cage. This distance between 3F windows (highlighted by the red planes in (c)) is 12.650(2) Å and between 2T1F windows (highlighted by the purple planes in (d)) is 10.555(3) Å.

Supplementary Note 3:

In calculating the T:F:MeOH:FA molar ratios of the methanol exchanged material (Figure S22), activated sample of $Zr_6(BDC)_3(Fum)_3$ (Figure S23) and the exact experimental formula of the activated sample, the quantities in which the two linkers, terephthalate and fumarate, were present was determined by ^{1}H NMR as described in Supplementary Note 1. Integrations were calculated relative to terephthalate, and the amount of formate and MeO^-/MeOH present was determined by the same method. As each formate molecule possesses just 1 proton, the value of the integration from the spectrum was multiplied by 4. Each methoxide possesses 3 protons, therefore, to calculate the quantity present, the value of the integration from the spectrum was divided by 3 and multiplied by 4. The maximum error of 2% established for relative ratios of peak integrals in the ^{1}H NMR spectrum[12] was considered when calculating the composition of the material.

The experimental formula of the activated sample of $Zr_6(BDC)_3(Fum)_3$, was calculated from the molar ratio of the four different species present which can act as ligands, terephthalate, fumarate, MeO^-/MeOH and formate, T:F:MeOH:FA = 1:0.92:0.6:0.2, obtained by ^{1}H NMR, and the assumption that the formula of the material should have the sum of the linkers is 6 or 24 coordinating sites per $[Zr_6O_4(OH)_4]^{12+}$cluster. Taking into account the 2% error on the value of the integration, this molar ratio has a maximum value of T:F:MeOH:FA = 1.02:0.94:0.61:0.2 and a minimum of T:F:MeOH:FA = 0.98:0.90:0.59:0.2. Due to the different binding modes of the ligands, the absence of a single terephthalate or fumarate linker leaves 4 coordination sites vacant at the $[Zr_6O_4(OH)_4]^{12+}$ cluster which can be occupied by two formates or two pairs of MeO^-/MeOH. Therefore we derived the following equation, $T + F + \left(\frac{FA}{2}\right) + \left(\frac{MeOH}{4}\right) = 6$, and used the above molar ratios to calculate the experimental formula of the framework composition. MeO^- and MeOH present is treated being present in equal quantities.

Calculation of Experimental Composition of $Zr_6(BDC)_3(Fum)_3$ by ^{1}H NMR:

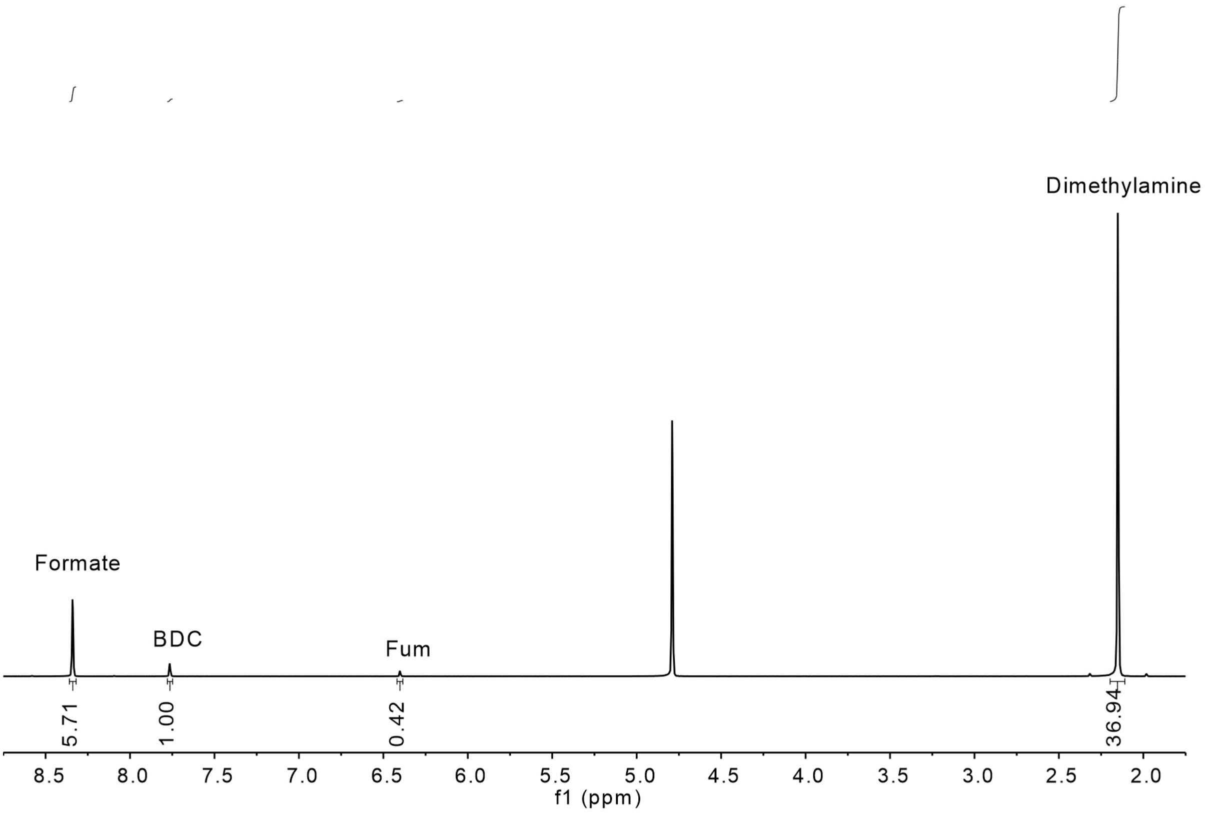

Figure S21 ^{1}H NMR spectrum of the crude product $Zr_6(BDC)_3(Fum)_3$, after washing with DMF and digestion in a mixture of NaOD (60 µL) and D_2O (640 µL). From the relative integrations of the terephthalate and fumarate peaks in the spectrum, the linkers are found to be present with a T:F ratio of T:F = 1:0.84. The observed fumarate deficiency from the nominal composition, T:F = 1:1, and lack of other species being present means that it is likely that some of the formate present is bound to the $[Zr_6O_4(OH)_4]^{12+}$ cluster at vacant binding sites, acting as a charge balancing species. However, the proportion of formate which can be attributed formate is bound to the cluster cannot be determined from this data, due to the presence of DMF in the pores and on the surface of the material. Under the conditions used for the digestion of the material, any DMF present is decomposed to dimethylamine and formate, giving rise to the two large peaks observed in the spectrum.

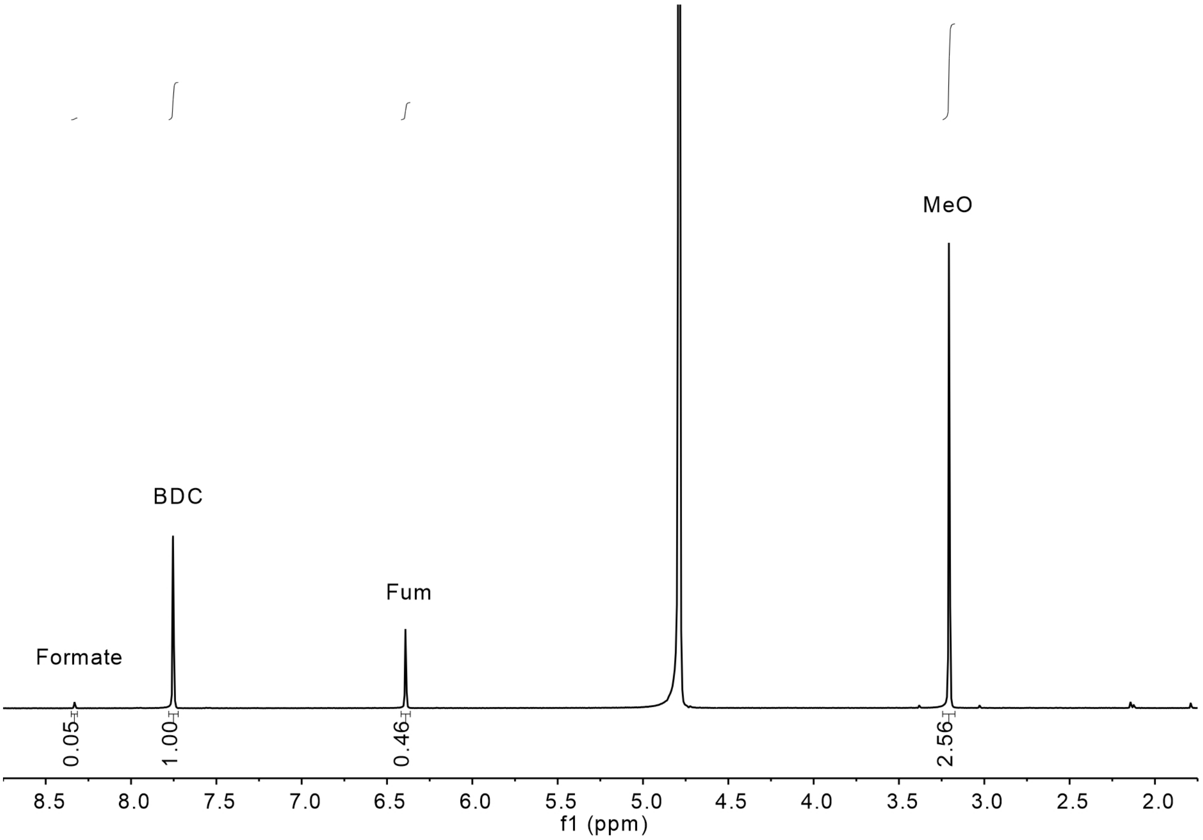

Figure S22 ^{1}H NMR spectrum of the sample of $Zr_6(BDC)_3(Fum)_3$, after solvent exchange with methanol. The material was dried under ambient conditions to allow most of the methanol on the surface of the material to evaporate prior to digestion in the NMR solvent mixture, NaOD (60 µL) and D_2O (640 µL). From the relative integrations of the terephthalate, fumarate, MeO^- /MeOH and formate peaks in the spectrum, the molar ratio in which these species are present was calculated as T:F:MeOH:FA = 1:0.92:3.4:0.2. The solvent exchange process successfully replaced the DMF in the pores of the material, as indicated by the lack of a dimethylamine peak in the NMR and the presence of a peak corresponding to methanol, which is present as methoxide under the conditions in which the ^{1}H NMR was run. The ratio between the two linkers T:F = 1:0.92 has changed compared to crude product, T:F = 1:0.84 because of the removal of unbound linkers by MeOH washing. The deviation in the equimolar ratio between the two linkers in the T:F:MeOH:FA molar ratio calculated from the spectrum, T:F:MeOH:FA = 1:0.92:3.4:0.2, indicates that there are missing linker defects in the structure, which results in vacant binding sites at the $[Zr_6O_4(OH)_4]^{12+}$ cluster. These vacant binding sites are occupied charge balancing species. One of these charge balancing species is formate, a small amount of which is still present in the ^{1}H NMR spectrum of the methanol exchanged sample, despite there being no dimethylamine. Therefore this remaining formate is likely to be bound to the $[Zr_6O_4(OH)_4]^{12+}$ clusters of the material, occupying missing linker defect sites. Some of these missing linker defect sites may also be occupied by methoxide, which can also fulfil the role of charge balancing. However, due to the presence of methanol in the pores and on the surface of the material, which by ^{1}H NMR cannot be distinguished from methoxide bound to the $[Zr_6O_4(OH)_4]^{12+}$ cluster, the number of missing linker defects and therefore exact composition of the material, cannot be accurately determined from the methanol exchanged sample.

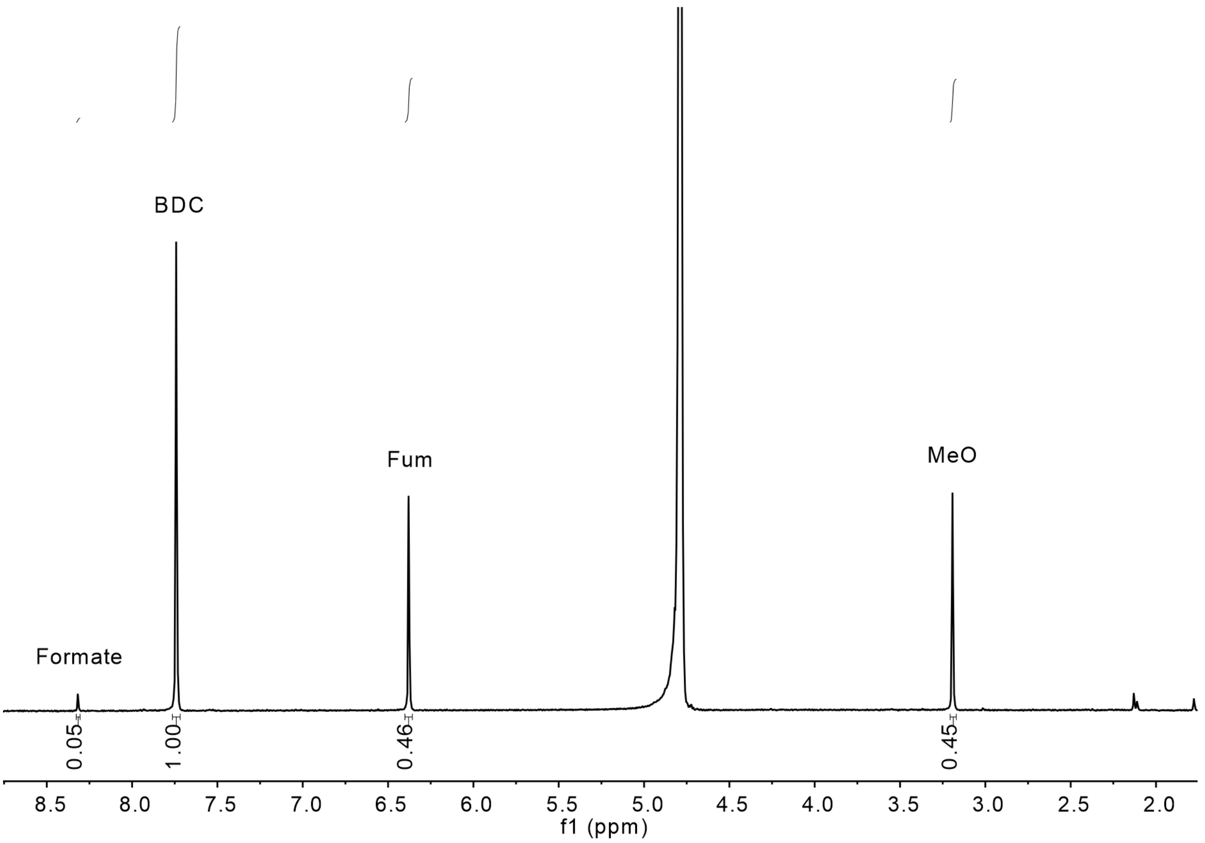

Figure S23 The ^{1}H NMR spectrum of $Zr_6(BDC)_3(Fum)_3$ obtained after solvent exchange with methanol, and activation under dynamic vacuum at 60 °C for 24 hours. This sample was used for the measurement of the N_2 adsorption desorption isotherm (Figure 8). From the relative integrations of the terephthalate, fumarate, MeO^{-}/MeOH and formate peaks in the spectrum, the molar ratio in which these species are present was calculated as T:F:MeOH:FA = 1:0.92:0.6:0.2. The process of activation under vacuum at elevated temperature removes all of the methanol from the pores and surface of the material, therefore meaning that all species observed in the ^{1}H NMR after activation are chemically bound to the structure and not simply guest species. This therefore means that the formate and methoxide present in the sample after activation can be attributed charge balancing species, bound to the $[Zr_6O_4(OH)_4]^{12+}$ cluster at vacant binding sites where the material exhibits defects, thus enabling the exact composition of the framework to be accurately calculated. The absence of a single linker leaves 4 binding sites vacant at the $[Zr_6O_4(OH)_4]^{12+}$ cluster, which can occupied by formate and pairs of MeO^{-}/MeOH. From the molar ratio in which terephthalate, fumarate, MeO^{-}/MeOH and formate are present in the sample, T:F:MeOH:FA = 1:0.92:0.6:0.2, and the theoretical formula of the material in which the sum of the linkers equates to 6, the formula of the activated material was calculated to be $Zr_6O_4(OH)_4(BDC)_{2.77}(Fum)_{2.55}(MeO)_{0.83}(MeOH)_{0.83}(Formate)_{0.55}$. This therefore means that, out of the theoretical 6 linkers, 0.68 (11.3%) are missing and these defect sites are occupied by formate and MeO^{-}/MeOH. This linker occupancy with 11.3% of missing linkers is in line with the values reported for single crystals of UiO-66, which contain 10% of missing linkers[5a, 5c], and powders of UiO-66 which contain up to 20% and 33% of missing linkers when prepared with a formic acid or trifluoroacetic acid modulators respectively.[5b] The formula calculated by ^{1}H NMR was consistent with the mass losses observed by TGA for the sample (Figure S25). Accounting for the 2% error associated with ^{1}H NMR the formula can be expressed as being within the range $Zr_6O_4(OH)_4(BDC)_{2.83}(Fum)_{2.60}(MeO)_{0.84}(MeOH)_{0.84}(Formate)_{0.56}$, and $Zr_6O_4(OH)_4(BDC)_{2.71}(Fum)_{2.49}(MeO)_{0.82}(MeOH)_{0.82}(Formate)_{0.54}$.

PXRD of MeOH Exchanged and Activated $Zr_6(BDC)_3(Fum)_3$:

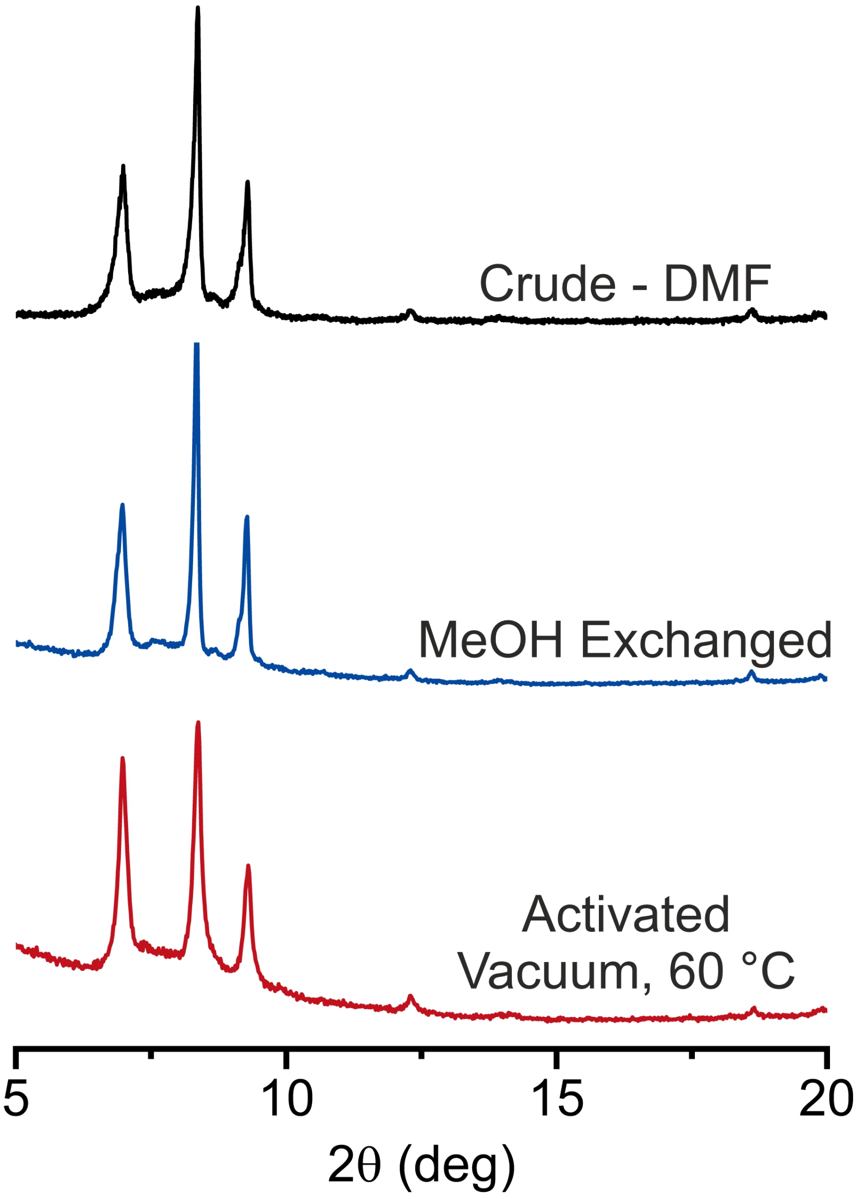

Figure S24 PXRD (Cu Kα_1, λ = 1.5406 Å) patterns of the sample of $Zr_6(BDC)_3(Fum)_3$, synthesised using a $ZrCl_4$ metal source, as the crude material in which the pores are filled with DMF (black), after solvent exchange with methanol (blue), and after the activation of the material (red) through removal of guest species from the pores under dynamic vacuum at 60 °C, 24 hours. The absence of any new peaks after the solvent exchange with methanol and the subsequent removal of the methanol guest species from the pores confirmed that $Zr_6(BDC)_3(Fum)_3$ retains its structure throughout the process of solvent exchange and activation. After activation, the composition of this sample was calculated by ^{1}H NMR (Figure S23) to be $Zr_6O_4(OH)_4(BDC)_{2.77}(Fum)_{2.55}(MeO)_{0.83}(MeOH)_{0.83}(Formate)_{0.55}$ and porosity measured by N_2 adsorption (Figure 8 and Table S11).

Thermogravimetric Analysis (TGA) of Activated $Zr_6(BDC)_3(Fum)_3$:

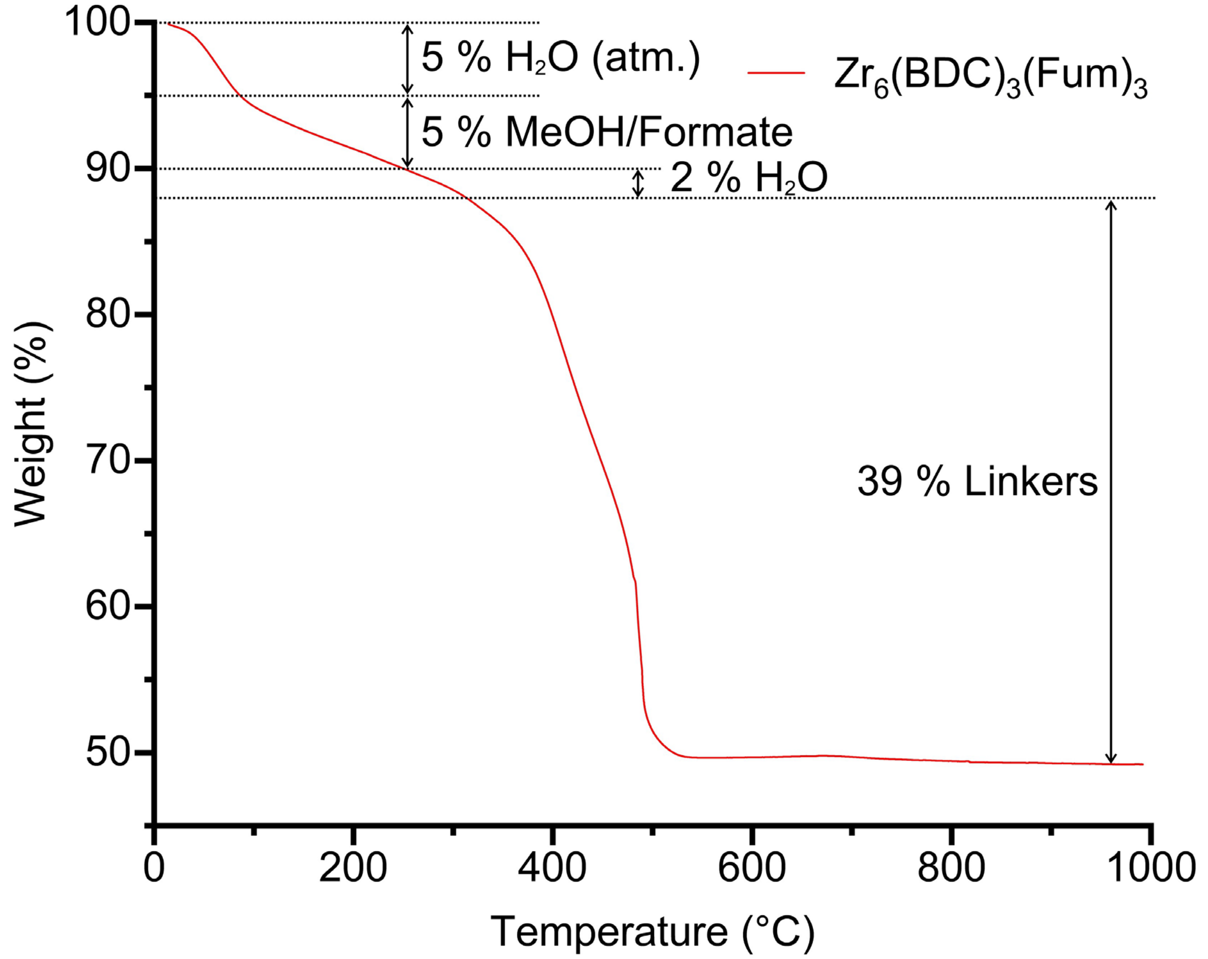

Figure S25 TGA plot for the sample of $Zr_6(BDC)_3(Fum)_3$, with the formula $Zr_6O_4(OH)_4(BDC)_{2.77}(Fum)_{2.55}(MeO)_{0.83}(MeOH)_{0.83}(Formate)_{0.55}$, activated under dynamic vacuum at 60 °C, under air at a flow rate of 100 mL min^{-1} with a heating rate of 10 °C min^{-1}. The initial mass loss of 5% up to 100 °C is attributed to loss of adsorbed atmospheric water from the material. The following mass losses were attributed to the components of $Zr_6(BDC)_3(Fum)_3$ considering that the formula presented above describes the 95% of the sample. The mass loss of 5% up to 225 °C is accounted for the expected loss of methanol, methoxide and formate from the material. This loss is followed by dehydroxylation of the clusters $[Zr_6O_4(OH)_4]^{12+}$ to $[Zr_6O_6]^{12+}$, with 2% mass loss. The thermal decomposition of the organic linkers that leaves ZrO_2 as residual corresponds to 39%. This is estimated by the complete mass loss of the two linkers and mass gain of six O atoms. The residual 49% mass is accounted for the amount of ZrO_2 produced from the complete decomposition of the starting material.

Elemental Analysis

Table S10 Elemental analysis for the sample of $Zr_6(BDC)_3(Fum)_3$. The theoretical values were calculated based on the formula obtained from ^{1}H NMR analysis $Zr_6O_4(OH)_4(BDC)_{2.77}(Fum)_{2.55}(MeO)_{0.83}(MeOH)_{0.83}(Formate)_{0.55}$.

	C (%)	H (%)
**Experimental**	27.18	1.85
**Theoretical**	27.65	1.78

Supplementary Note 4:

The experimental values for the BET surface area and pore volume of MOF-801 and UiO-66 MOFs were taken from Furukawa et al.[8a], while the values for our material were obtained following the protocols described in section Methods. The calculated values were obtained using Zeo++[13] for a spherical probe of 3.64 Å in diameter (the kinetic diameter of a dinitrogen molecule) for reported CIF files of the three MOFs. To standardise the description of the $[Zr_6O_4(OH)_4]^{12+}$ cluster in all three structures, all μ_3-O(H) bridges were treated as equivalent to each other and represented by a single oxygen atom located at the average position of the two oxygen atoms. The calculated values in Table S11 were obtained using the calculated density of an ideal defect-free structure. All calculated values are very much in line with the experimental observations.

Porosity Measurement Data:

Table S11: Comparison of the calculated (black) and experimental (red) values of the BET surface area and pore volume of $Zr_6(BDC)_3(Fum)_3$ with those of UiO-66 and MOF-801.[8a] The N_2 adsorption isotherm was recorded for the activated sample with the formula $Zr_6O_4(OH)_4(BDC)_{2.77}(Fum)_{2.55}(MeO)_{0.83}(MeOH)_{0.83}(Formate)_{0.55}$ as calculated by ^1H NMR (Figure S23) and which retains its structure by PXRD after activation (Figure S24). $Zr_6(BDC)_3(Fum)_3$ has an experimental BET surface area of 783 m^2g^{-1} and a pore volume of 0.32 cm^3g^{-1}. These are slightly larger than the theoretical calculated values of 714 m^2g^{-1} and 0.26 cm^3g^{-1} respectively, calculated using Zeo++[13] (Supplementary Note 4). The calculated densities of an ideal defect-free structure are also given for each material.

	**MOF-801**	**$Zr_6(BDC)_3(Fum)_3$**	**UiO-66**
**Experimental BET surface area, m^2/g**	690	783	1290
**Calculated surface area, m^2/g**	599	714	1124
**Experimental pore volume, cm^3/g**	0.27	0.32	0.49
**Calculated pore volume, cm^3/g**	0.24	0.26	0.37
**Calculated density, g/cm^3**	1.597	1.425	1.238

PXRD $Zr_6(BDC)_3(Fum)_3$ in Water

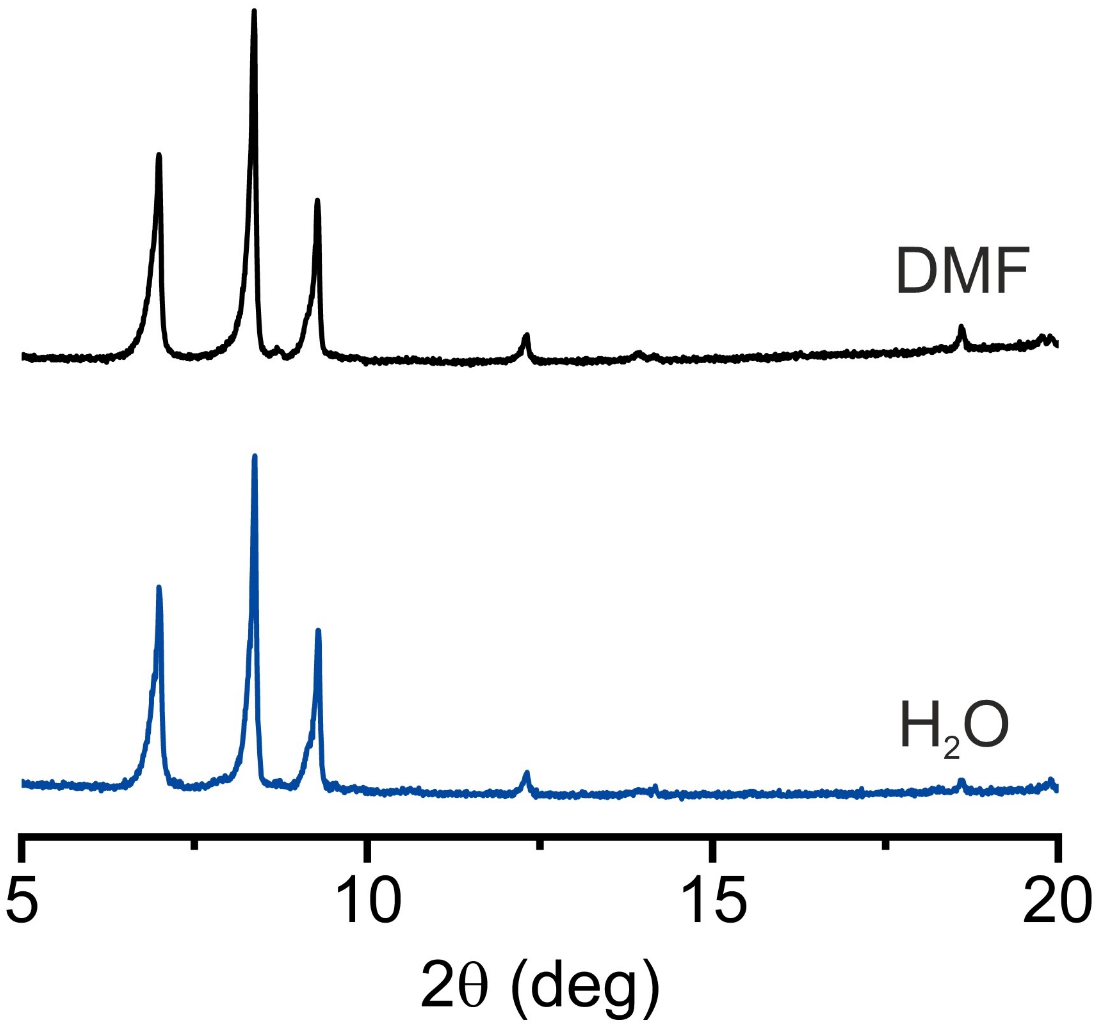

Figure S26 PXRD (Cu Kα_1, λ = 1.5406 Å) patterns of a sample of $Zr_6(BDC)_3(Fum)_3$ as the crude material which was recorded in DMF (black) and after 3 days of immersed in water (blue). The absence of any new peaks or changes in the relative intensities in the pattern recorded in indicates that the structure is retained and that $Zr_6(BDC)_3(Fum)_3$ is water stable.

References:

[1] A. A. Coelho, *Journal of Applied Crystallography* **2003**, *36*, 86-95.
[2] A. A. Coelho, in *TOPAS Academic V5*, Coelho Software, **2012**.
[3] R. A. Young, *The Rietveld Method*, Oxford University Press, New York, USA, **1981**.
[4] R. W. Cheary, A. A. Coelho, *Journal of Applied Crystallography* **1992**, *25*, 109-121.
[5] a) L. Liu, Z. Chen, J. Wang, D. Zhang, Y. Zhu, S. Ling, K.-W. Huang, Y. Belmabkhout, K. Adil, Y. Zhang, B. Slater, M. Eddaoudi, Y. Han, *Nature Chemistry* **2019**, *11*, 622-628; b) G. C. Shearer, S. Chavan, S. Bordiga, S. Svelle, U. Olsbye, K. P. Lillerud, *Chemistry of Materials* **2016**, *28*, 3749-3761; c) C. A. Trickett, K. J. Gagnon, S. Lee, F. Gándara, H.-B. Bürgi, O. M. Yaghi, *Angewandte Chemie International Edition* **2015**, *54*, 11162-11167; d) H. Wu, Y. S. Chua, V. Krungleviciute, M. Tyagi, P. Chen, T. Yildirim, W. Zhou, *Journal of the American Chemical Society* **2013**, *135*, 10525-10532.
[6] S. Øien, D. Wragg, H. Reinsch, S. Svelle, S. Bordiga, C. Lamberti, K. P. Lillerud, *Crystal Growth & Design* **2014**, *14*, 5370-5372.
[7] a) O. V. Gutov, M. G. Hevia, E. C. Escudero-Adán, A. Shafir, *Inorganic Chemistry* **2015**, *54*, 8396-8400; b) W. Morris, S. Wang, D. Cho, E. Auyeung, P. Li, O. K. Farha, C. A. Mirkin, *ACS Applied Materials & Interfaces* **2017**, *9*, 33413-33418.
[8] a) H. Furukawa, F. Gándara, Y.-B. Zhang, J. Jiang, W. L. Queen, M. R. Hudson, O. M. Yaghi, *Journal of the American Chemical Society* **2014**, *136*, 4369-4381; b) F. Ke, C. Peng, T. Zhang, M. Zhang, C. Zhou, H. Cai, J. Zhu, X. Wan, *Scientific Reports* **2018**, *8*, 939; c) H. Kim, S. R. Rao, E. A. Kapustin, L. Zhao, S. Yang, O. M. Yaghi, E. N. Wang, *Nature Communications* **2018**, *9*, 1191; d) H. Motegi, K. Yano, N. Setoyama, Y. Matsuoka, T. Ohmura, A. Usuki, *Journal of Porous Materials* **2017**, *24*, 1327-1333.
[9] L. Valenzano, B. Civalleri, S. Chavan, S. Bordiga, M. H. Nilsen, S. Jakobsen, K. P. Lillerud, C. Lamberti, *Chemistry of Materials* **2011**, *23*, 1700-1718.
[10] G. Wißmann, A. Schaate, S. Lilienthal, I. Bremer, A. M. Schneider, P. Behrens, *Microporous and Mesoporous Materials* **2012**, *152*, 64-70.
[11] a) C. B. Barber, D. P. Dobkin, H. Huhdanpaa, *ACM Trans. Math. Softw.* **1996**, *22*, 469–483; b) P. Virtanen, R. Gommers, T. E. Oliphant, M. Haberland, T. Reddy, D. Cournapeau, E. Burovski, P. Peterson, W. Weckesser, J. Bright, S. J. van der Walt, M. Brett, J. Wilson, K. J. Millman, N. Mayorov, A. R. J. Nelson, E. Jones, R. Kern, E. Larson, C. J. Carey, İ. Polat, Y. Feng, E. W. Moore, J. VanderPlas, D. Laxalde, J. Perktold, R. Cimrman, I. Henriksen, E. A. Quintero, C. R. Harris, A. M. Archibald, A. H. Ribeiro, F. Pedregosa, P. van Mulbregt, A. Vijaykumar, A. P. Bardelli, A. Rothberg, A. Hilboll, A. Kloeckner, A. Scopatz, A. Lee, A. Rokem, C. N. Woods, C. Fulton, C. Masson, C. Häggström, C. Fitzgerald, D. A. Nicholson, D. R. Hagen, D. V. Pasechnik, E. Olivetti, E. Martin, E. Wieser, F. Silva, F. Lenders, F. Wilhelm, G. Young, G. A. Price, G.-L. Ingold, G. E. Allen, G. R. Lee, H. Audren, I. Probst, J. P. Dietrich, J. Silterra, J. T. Webber, J. Slavič, J. Nothman, J. Buchner, J. Kulick, J. L. Schönberger, J. V. de Miranda Cardoso, J. Reimer, J. Harrington, J. L. C. Rodríguez, J. Nunez-Iglesias, J. Kuczynski, K. Tritz, M. Thoma, M. Newville, M. Kümmerer, M. Bolingbroke, M. Tartre, M. Pak, N. J. Smith, N. Nowaczyk, N. Shebanov, O. Pavlyk, P. A. Brodtkorb, P. Lee, R. T. McGibbon, R. Feldbauer, S. Lewis, S. Tygier, S. Sievert, S. Vigna, S. Peterson, S. More, T. Pudlik, T. Oshima, et al., *Nature Methods* **2020**, *17*, 261-272.
[12] S. K. Bharti, R. Roy, *TrAC Trends in Analytical Chemistry* **2012**, *35*, 5-26.
[13] T. F. Willems, C. H. Rycroft, M. Kazi, J. C. Meza, M. Haranczyk, *Microporous and Mesoporous Materials* **2012**, *149*, 134-141.
